# Peculiar combinations of individually non-pathogenic missense mitochondrial DNA variants cause low penetrance Leber’s hereditary optic neuropathy

**DOI:** 10.1371/journal.pgen.1007210

**Published:** 2018-02-14

**Authors:** Leonardo Caporali, Luisa Iommarini, Chiara La Morgia, Anna Olivieri, Alessandro Achilli, Alessandra Maresca, Maria Lucia Valentino, Mariantonietta Capristo, Francesca Tagliavini, Valentina Del Dotto, Claudia Zanna, Rocco Liguori, Piero Barboni, Michele Carbonelli, Veronica Cocetta, Monica Montopoli, Andrea Martinuzzi, Giovanna Cenacchi, Giuseppe De Michele, Francesco Testa, Anna Nesti, Francesca Simonelli, Anna Maria Porcelli, Antonio Torroni, Valerio Carelli

**Affiliations:** 1 Neurology Unit, IRCCS Institute of Neurological Sciences of Bologna, Bologna, Italy; 2 Department of Pharmacy and Biotechnology (FABIT), University of Bologna, Bologna, Italy; 3 Department of Biomedical and NeuroMotor Sciences (DIBINEM), University of Bologna, Bologna, Italy; 4 Department of Biology and Biotechnology "L. Spallanzani", University of Pavia, Pavia, Italy; 5 Studio Oculistico D’Azeglio, Bologna, Italy; 6 Department of Pharmaceutical and Pharmacological Sciences, University of Padova, Padua, Italy; 7 IRCCS "E. Medea" Scientific Institute Conegliano-Pieve di Soligo Research Center, Pieve di Soligo, Italy; 8 Department of Neuroscience, Reproductive Sciences and Dentistry, University of Naples “Federico II”, Naples, Italy; 9 Eye Clinic, Multidisciplinary Department of Medical, Surgical and Dental Sciences, University of Campania “Luigi Vanvitelli”, Naples, Italy; 10 Health Sciences & Technologies (HST) CIRI, University of Bologna, Bologna, Italy; Max Planck Institute for Biology of Ageing, GERMANY

## Abstract

We here report on the existence of Leber’s hereditary optic neuropathy (LHON) associated with peculiar combinations of individually non-pathogenic missense mitochondrial DNA (mtDNA) variants, affecting the *MT-ND4*, *MT-ND4L* and *MT-ND6* subunit genes of Complex I. The pathogenic potential of these mtDNA haplotypes is supported by multiple evidences: first, the LHON phenotype is strictly inherited along the maternal line in one very large family; second, the combinations of mtDNA variants are unique to the two maternal lineages that are characterized by recurrence of LHON; third, the Complex I-dependent respiratory and oxidative phosphorylation defect is co-transferred from the proband’s fibroblasts into the cybrid cell model. Finally, all but one of these missense mtDNA variants cluster along the same predicted fourth E-channel deputed to proton translocation within the transmembrane domain of Complex I, involving the ND1, ND4L and ND6 subunits. Hence, the definition of the pathogenic role of a specific mtDNA mutation becomes blurrier than ever and only an accurate evaluation of mitogenome sequence variation data from the general population, combined with functional analyses using the cybrid cell model, may lead to final validation. Our study conclusively shows that even in the absence of a clearly established LHON primary mutation, unprecedented combinations of missense mtDNA variants, individually known as polymorphisms, may lead to reduced OXPHOS efficiency sufficient to trigger LHON. In this context, we introduce a new diagnostic perspective that implies the complete sequence analysis of mitogenomes in LHON as mandatory gold standard diagnostic approach.

## Author summary

Leber’s hereditary optic neuropathy (LHON) is a common cause of maternally inherited vision loss. In the large majority of cases LHON is due to mitochondrial DNA (mtDNA) point mutations, clearly distinct from common polymorphisms normally found in the general population, affecting the mitochondrial function, thus defined as pathogenic. For the first time, we here demonstrate, on the genetic and functional ground, that unusual combinations of otherwise polymorphic and non-pathogenic mtDNA variants are sufficient for causing low-penetrance maternally inherited optic neuropathy in pedigrees fitting the LHON clinical diagnosis. Our findings bridge the blurry border between “pathogenic” and “neutral” mutations in an overall continuum that truly depends on the specific and sometime unique combination of variants characterizing each mitogenome. As a result, we conclude that, for an accurate diagnosis of LHON and possibly of other mitochondrial diseases, the only approach that can disclose all possible causative sources is complete mitogenome sequencing.

## Introduction

Since the identification of the first causal mitochondrial DNA (mtDNA) point mutation [1], the mutational landscape of Leber’s hereditary optic neuropathy (LHON) has become increasingly complex. In particular, LHON pathogenic mutations are frequently homoplasmic and, in some cases, their pathogenicity has not been readily recognized.[2][3] Now we know that over 90% of LHON patients are due to three common mtDNA point mutations m.11778G>A/*MT-ND4*, m.3460G>A/*MT-ND1* and m.14484T>C/*MT-ND6* [4][5].

Interestingly, the pathogenic role of the m.14484T>C/*MT-ND6* was initially not recognized because of the low phylogenetic conservation of the affected amino acid [6][7]. Later, it became also clear that some mtDNA variants, classified as secondary mutations, were associated with LHON, despite being present at high frequencies in control populations [8,9][10]. The debate on this issue [11,12] was resolved by recognizing these variants as markers of specific mtDNA haplogroups [13,14], and showing that two clades of the western Eurasian haplogroup J were genetic backgrounds enhancing the pathogenic potential of the m.14484T>C/*MT-ND6*, and at a weaker extent the m.11778G>A/*MT-ND4* mutations [15,16]. As counterproof of this scenario, the m.14484T>C/*MT-ND6* change found on non-J mtDNA backgrounds displays a very low penetrance and has been occasionally reported in genetic surveys of control populations, thus behaving borderline and similarly to a polymorphic variant [17,18].

More recently, *MT-ND6* and *MT-ND1* have been highlighted as LHON mutational hotspots [19,20], since multiple rare LHON pathogenic mutations, often preferentially associated with haplogroup J, have been reported to affect these genes [21]. Furthermore, different sets of two or more mtDNA variants have been postulated as modulators of penetrance, such as combinations of multiple private “weak” pathogenic mutations or combinations of established LHON pathogenic mutations with variants, already known as markers of specific haplogroups, but detected outside the usual haplogroup background [22,23]. Similar conclusions have been reached in the context of East Asian haplogroups by complete mtDNA sequencing of Asian LHON pedigrees, as compared with population-matched controls [24–27]. Thus, the identification of truly pathogenic variants, distinguished from synergistic modifying variants in various combinations, is increasingly challenging.

We here present evidence that unusual combinations of otherwise polymorphic and non-pathogenic mtDNA missense mutations may be sufficient for causing low-penetrance maternally inherited optic neuropathy fitting the LHON clinical diagnosis in independent pedigrees. Our findings bridge the blurry border between “pathogenic” and “neutral” mutations in an overall continuum that truly depends on the specific and sometime unique combination of variants characterizing each mitogenome.

## Results

### Pedigrees investigated

**Fig 1:**
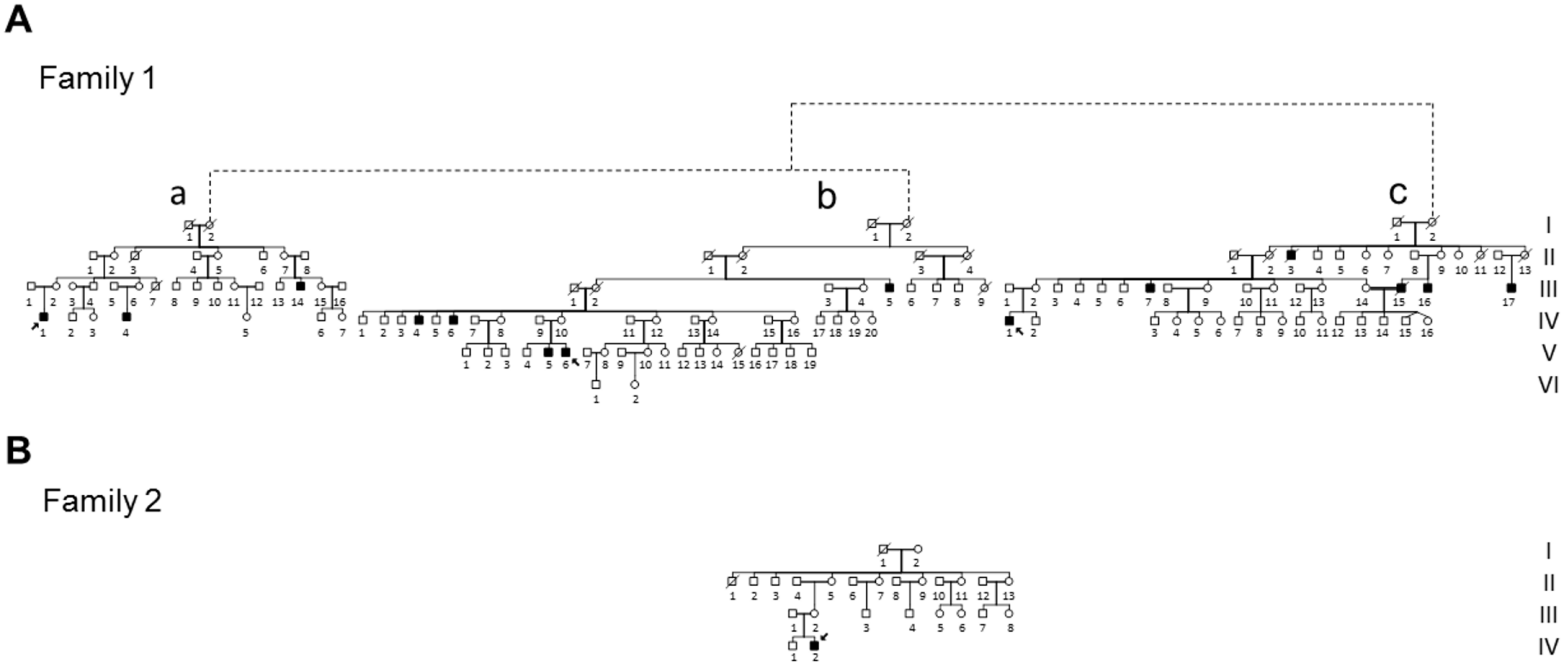
Pedigrees. **A.** Pedigree of Family 1 with the reconstructed genealogy (indicated by dashed lines) of its three branches (a, b, c). Affected individuals are indicated in black; probands are indicated by arrows.**B.** Pedigree of Family 2. Affected individuals are indicated in black; proband is indicated by arrow.

**Fig 2:**
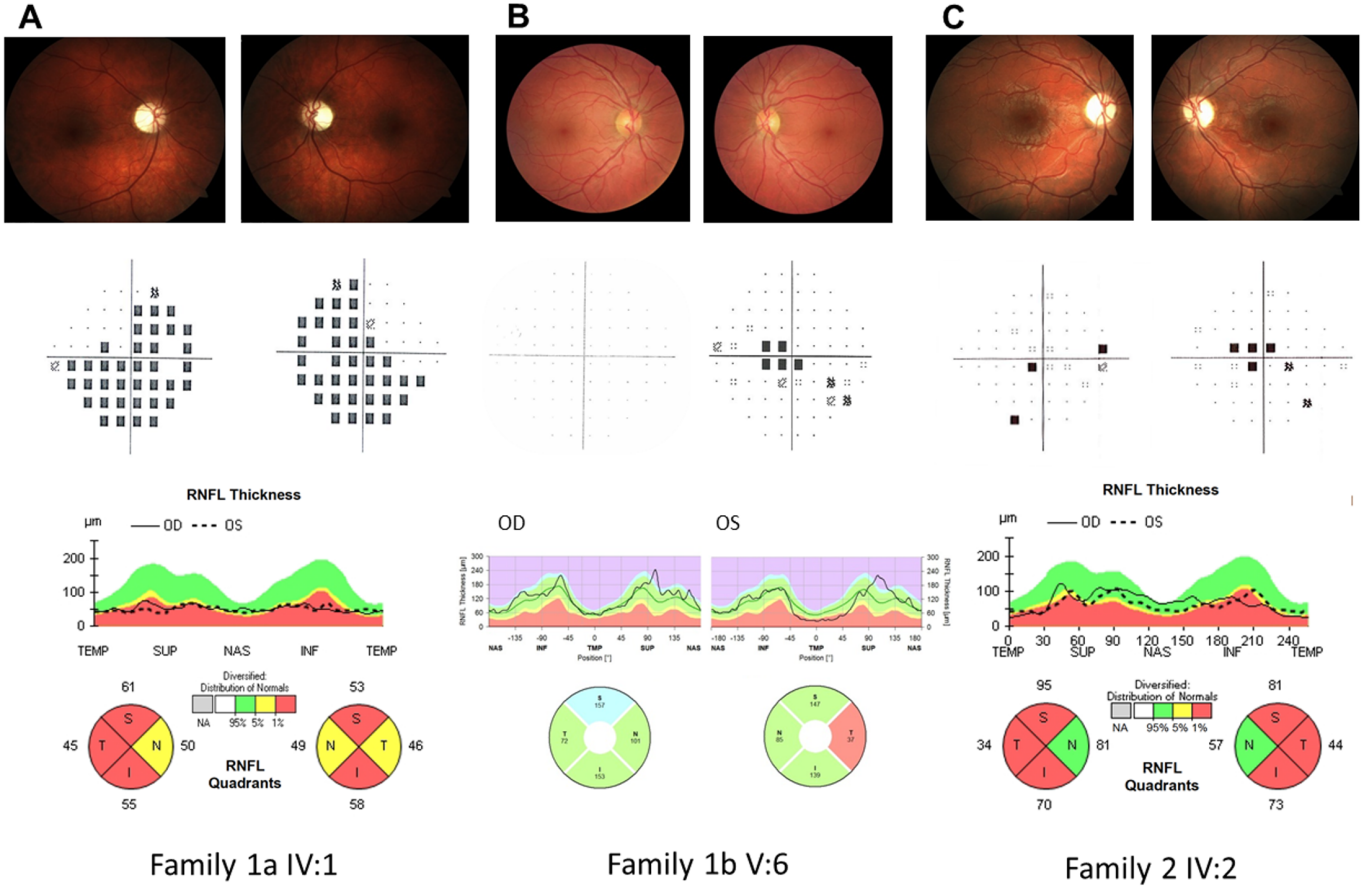
Ophthalmological data. **A. Family 1a IV:1** Upper line: Fundus oculi pictures show bilateral optic nerve pallor. Middle line: Computerized visual fields (VFs) (pattern deviation) reveal bilateral generalized defect. Lower line: Optical coherence tomography (OCT) (Cirrus, Zeiss) demonstrates bilateral diffuse optic atrophy with relative nasal sparing. **B. Family 1b V:6** Upper line: Fundus oculi pictures show normal optic nerve in OD and mild temporal pallor in OS. Middle line: Computerized VFs is unremarkable in OD and demonstrates a small central scotoma in OS. Lower line: OCT demonstrates normal retinal nerve fiber layer thickness in OD and temporal thinning in OS (Spectralis, Heidelberg). **C. Family 2 IV:2** Upper line: Fundus oculi pictures show bilateral optic nerve pallor. Middle line: Computerized VFs reveal bilateral central scotoma. Lower line: OCT demonstrates bilateral diffuse optic atrophy with relative nasal sparing (Cirrus, Zeiss).

We observed three multigenerational pedigrees (Families 1a, b, and c in Fig 1A) with multiple affected individuals fitting the clinical diagnosis of LHON and with a clear maternal transmission of the phenotype. Noticeably, all three pedigrees were from the same geographical area of southern Italy (Campania region). A fourth smaller pedigree (Family 2; Fig 1B) from northern Italy (Emilia-Romagna region) was observed with a single affected individual obeying the LHON clinical diagnosis. All four families tested negative for the three common LHON mutations at positions m.11778G>A/*MT-ND4*, m.3460G>A/*MT-ND1* and m.14484T>C/*MT-ND6*. Clinical histories of affected individuals are reported in detail in Supporting Information (S1 Text, S1 and S2 Tables). Some examples of ophthalmological features are illustrated in Fig 2.

### Skeletal muscle investigations reveal normal activity of respiratory chain complexes but increased mitochondrial biogenesis

**Fig 3:**
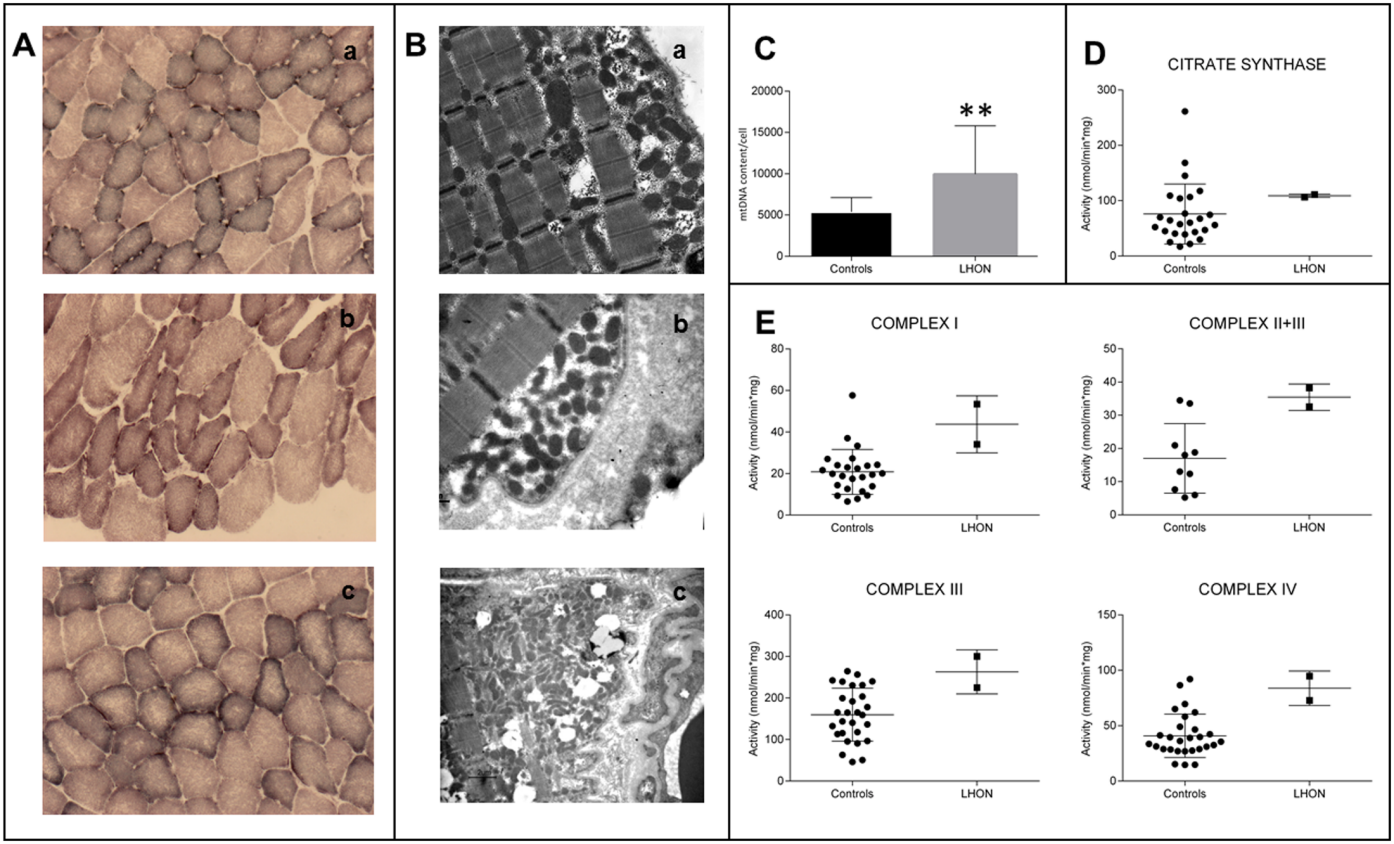
Morphological, molecular and biochemical analysis on skeletal muscle biopsies. **A.** SDH staining of skeletal muscle biopsies from individuals IV:1 from Family 1a (a), V:6 from Family 1b (b) and IV:2 from Family 2 (c). **B**. Transmission electron microscopy of the same muscle specimens as in A. Both SDH histoenzymatic staining (A) and ultrastructural evaluation (B) demonstrate mitochondrial proliferation as highlighted respectively by subsarcolemmal increase of SDH reaction and corresponding accumulation of mitochondria. **C.** Assessment of mtDNA content per cell, presented as column with mean ± SD (*n* = 3; **p<0.001), confirms the activation of mitochondrial biogenesis as shown by the significant increase in LHON samples. **D.** The evaluation of citrate synthase activity, presented as scatter plot with mean ± SD, parallels again the results observed in C, with an increased mean value in the LHON samples. **E.** Evaluations of Complex I, Complex II+III, Complex III, Complex IV activities, presented as scatter plot with mean ± SD, reveal an increase of all activities in LHON samples.

Histological analysis of skeletal muscle biopsies from the probands of Families 1a, 1b and 2 (Fig 1) showed no overt signs of myopathy with minimal variability in fibers size, and histoenzymatic stain showed normal COX activity, but some increase of subsarcolemmal SDH reactivity (Fig 3A). TEM analysis confirmed the presence of proliferated mitochondria under the sarcolemma and, occasionally, between fibers (Fig 3B). Mitochondrial DNA copy number and citrate synthase (CS) activity were both increased in skeletal muscles of patients as compared to controls (Fig 3C and 3D). Similarly, the specific oxidoreductase activities of Complex I (CI), Complex II+III (CII+III), Complex III (CIII) and Complex IV (CIV) were increased (Fig 3E), whereas they were comparable to controls when normalized on CS activity (S3 Table). Taken together these data indicate the occurrence of an activated compensatory mitochondrial biogenesis, most likely due to a compensatory response caused by a mild mitochondrial defect, as previously reported [28,29].

### Molecular investigations, protein conservation, frequency, and phylogenetic analyses of the identified variants

**Fig 4:**
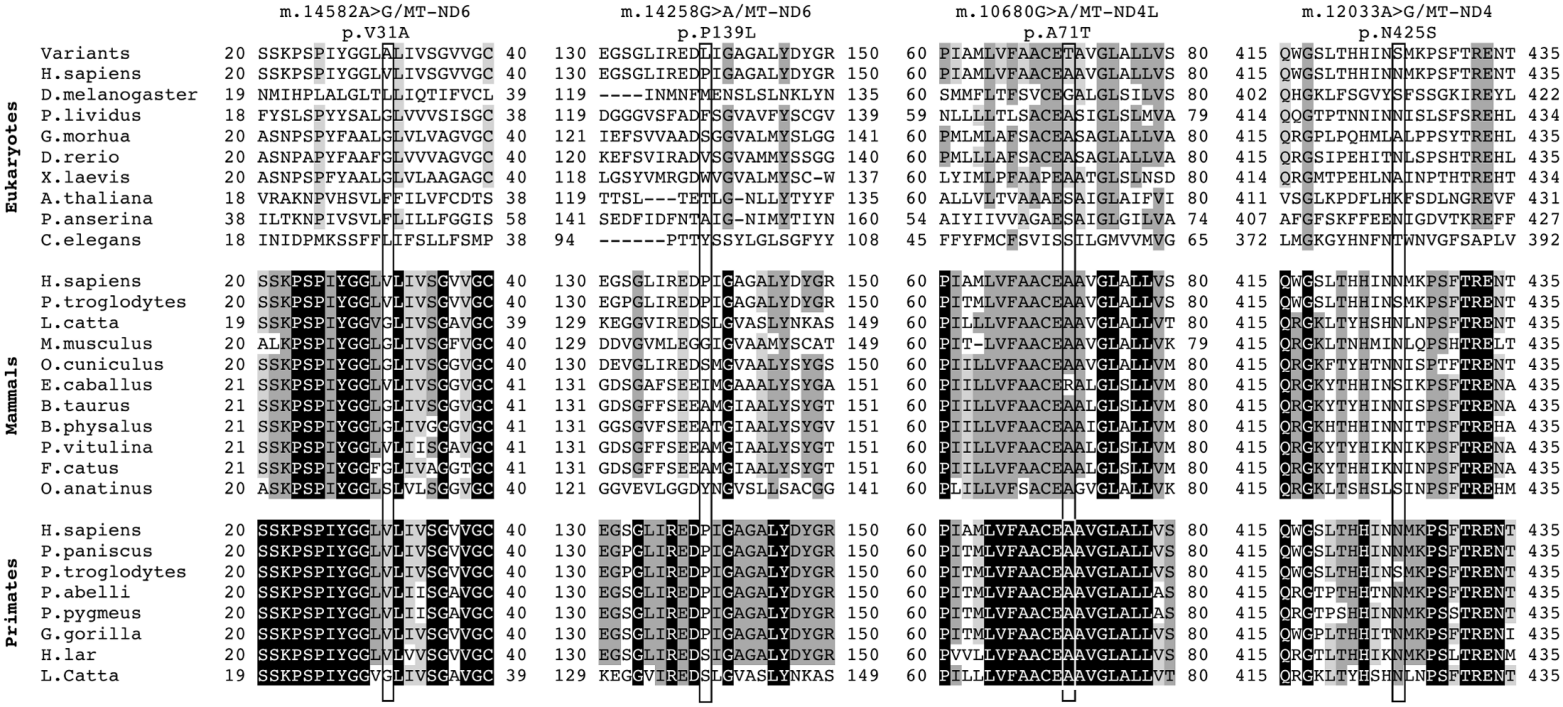
Amino acid conservation analysis. Global alignment of ND1, ND4L and ND6 protein sequences from a wide range of eukaryotes, mammals and primates. The neighborhoods (20 amino acids) of m.14582A>G/*MT-ND6*, m.14258G>A/*MT-ND6*, m.10680G>A/*MT-ND4L* and m.12033A>G/*MT-ND4* are shown. Rectangles frame these specific variants. Amino acid residues with a percentage of conservation ranging between 70.0% and 79.9% are highlighted in light grey, those between 80.0% and 99.9% are highlighted in dark grey and those invariant (100%) are highlighted in black.

Sequencing of the entire mitogenome from each of the probands independently ascertained for Families 1a, 1b and 1c revealed the same identical sequence, indicating that they descend from the same maternal ancestor, as also suggested by their geographical proximity. They all shared the diagnostic variants for haplogroup K1a and the following private mutations: non-coding m.2281A>G/*MT-RNR2* and m.16129G>A/*MT-HV1*; synonymous m.6137T>C/*MT-CO1*, m.6329C>T/*MT-CO1*, m.8994G>A/*MT-ATP6*, m.11038A>G/*MT-ND4* and m.15253A>G/*MT-CYB*; and missense m.14258G>A/*MT-ND6* and m.14582A>G/*MT-ND6*. The m.14258G>A/*MT-ND6* mutation causes the amino acid substitution p.P139L, and the m.14582A>G/*MT-ND6* the amino acid substitution p.V31A. Both missense mutations affect poorly conserved positions of the ND6 subunit of CI and are not predicted to be damaging (Fig 4, S4 Table). However, the P139 position showed a higher conservation degree in mammals (37%) compared to eukaryotes (22%), being highest in primates (66%). Furthermore, the P139 position in mammals sits two residues away from an invariant position (G141) and, in primates is contiguous to another invariant position (D138) within a moderately conserved domain (6 invariant positions out of 21) (Fig 4). The V31 position shows similar features. In eukaryotes and mammals glycine is prevalent at position 31 with a low conservation (49% and 46%, respectively). In primates, at this position valine becomes prevalent with a higher conservation (77%). Most relevantly, V31 is within a highly conserved domain (10 invariant positions out of 21) in mammals, and is even more conserved in primates (16 invariant positions out of 21) (Fig 4).

Complete mtDNA sequence analysis of the proband from Family 2 showed all the variants diagnostic for haplogroup H5b and the following private mutations: synonymous m.10248T>C/*MT-ND3*; missense m.9966G>A/*MT-CO3*, m.10680G>A/*MT-ND4L*, m.12033A>G/*MT-ND4* and m.14258G>A/*MT-ND6*. Besides the m.14258G>A/*MT-ND6* nucleotide change, Family 2 also harbored the m.10680G>A/*MT-ND4L* and the m.12033A>G/*MT-ND4* mutations that induce the amino acid changes p.A71T in ND4L and the p.N425S in ND4 subunits of CI, respectively. The p.A71T change affects a highly conserved ND4L position (86% in eukaryotes, 97% in mammals, invariant in primates), within an invariant stretch of 16 amino acids in primates (Fig 4, S4 Table). Conversely, the p.N425S affects a highly conserved ND4 position only in primates (41% in eukaryotes, 70% in mammals, 87% in primates), in a moderately conserved domain (9 invariant positions out of 21) (Fig 4, S4 Table). Both variants were considered neutral for the protein function by most of the prediction tools employed.

The m.14258G>A/*MT-ND6* change, found in both Families 1 and 2, has been previously reported according to Mitomap and HmtDB in 10 different haplogroups, being diagnostic for haplogroups U3a1a1 and H1q3. The m.14582A>G/*MT-ND6* (Family 1) variant has been previously reported in seven different haplogroups, being diagnostic for haplogroup H4a. In these databases, the sample classified as GenBank: KC878720 is from our Family 1 and it was previously published [30] without considering the recurrence of a clinical phenotype (A. Torroni, personal communication). The coexistence of m.14258G>A/*MT-ND6* with the m.14582A>G/*MT-ND6* variants is, however, unique to Family 1, when compared to all the other reported cases (S5 Table).

Concerning the m.10680G>A/*MT-ND4L* variant, this has been found in 14 haplogroups and it has been previously reported as the only pathogenic change in three LHON families, arising as independent mutational events in haplogroups B4a1e, M13a1b and D6a1 [31,32]. In addition, this mutation has also been found in association with the m.14484T>C/*MT-ND6* mutation in a further LHON family with a haplogroup B4d1 background [26]. However, the m.10680G>A/*MT-ND4L* change has also been recognized in ten different maternal lineages with no pathology reported (S6 Table).

Finally, the m.12033A>G/*MT-ND4* variant has been reported in five different haplogroups in the general population, without being associated with any pathologic phenotype. Overall, the combination of the three coexisting missense changes m.10680G>A/*MT-ND4L*, m.12033A>G/*MT-ND4* and m.14258G>A/*MT-ND6* is a unique feature of Family 2.

We also screened by complete mtDNA sequencing our entire cohort of LHON probands with one of the three known primary mutations (*n* = 236), finding the m.14258G>A/*MT-ND6* variant in two further families, carrying the m.11778G>A /*MT-ND4* (haplogroup T1a1) and m.14484T>C/*MT-ND6* (haplogroup L2a1a1) LHON mutations, respectively (Families 3 and 4; S1 and S2 Figs).

In Families 1a-b-c all available individuals along the maternal lines (*n* = 22) were RFLP surveyed for the m.14258G>A/*MT-ND6* and m.14582A>G/*MT-ND6* variants, which appeared always homoplasmic. In Family 2, the proband’s mother and sister were also homoplasmic for all missense variants (m.10680G>A/*MT-ND4L*, m.12033A>*G/MT-ND4* and m.14258G>A/*MT-ND6*).

### Cybrid studies

**Fig 5:**
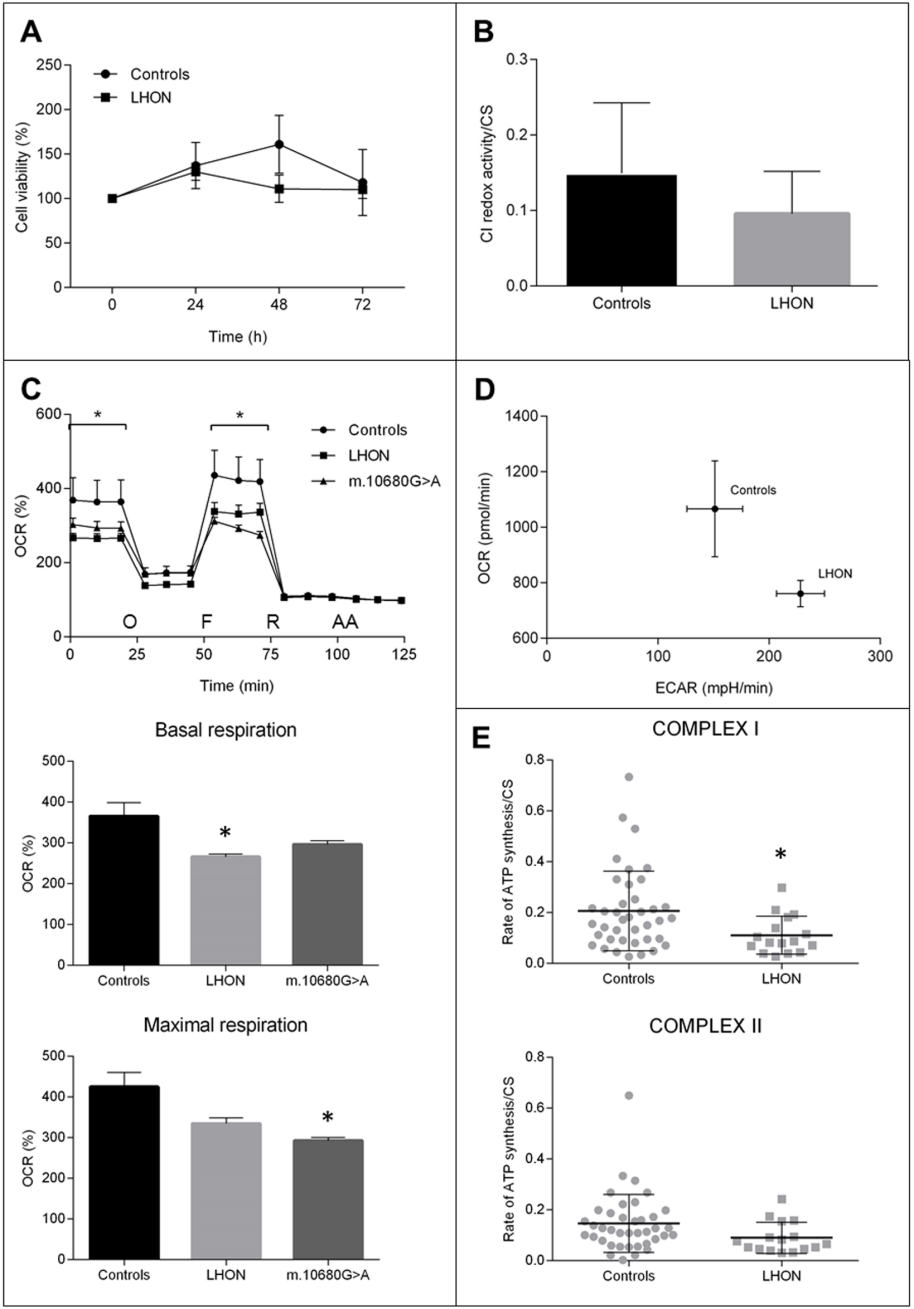
Biochemical characterization of cybrid clones. **A.** Cell viability after different time of incubation in galactose medium (0, 24, 48, 72h). Data are expressed as percentage of T0 (*n* = 12; mean ± SEM). **B.** Rotenone sensitive redox activity of respiratory complex I normalized for CS activity (*n* = 9; mean ± SD). **C.** OCR traces as pmol O_2_/min, after the injection of 1μM oligomycin (O), 0.2μM FCCP (F), 1μM rotenone (R) and 1μM antimycin A (AA) (mean ± SEM). Asterisks indicate statistical significance (*n>* = 3; * p<0.05). **D.** XFe Metabolic Phenogram. Basal OCR (pmol/min) and ECAR (mpH/min) rates were plotted in controls vs LHON cybrids, showing a metabolic shift in LHON cybrids towards glycolysis. **E.** ATP synthesis rates normalized for CS activity driven by complex I substrates (malate/glutamate) and complex II substrate succinate (mean ± SD). Asterisks indicate statistical significance (*n* = 16; * p<0.05).

To assess the pathogenic potential of the two peculiar combinations of missense variants found in Family 1 (m.14258G>A/*MT-ND6* and m.14582A>G/*MT-ND6*) and Family 2 (m.10680G>A/*MT-ND4L*, m.12033A>G/*MT-ND4* and m.14258G>A/*MT-ND6*) we generated cybrids using enucleated fibroblasts derived from the probands of Families 1a, 1b and 2, as cytoplast donors. As detailed in Material and Methods, different cell clones were obtained harboring each the two combinations of homoplasmic variants and used for subsequent investigations. In Fig 5, the data obtained from each LHON cell line were pooled together and compared to control cybrids, as they proliferate at similar rates in complete medium (25 mM glucose) (S3 Fig). To challenge the mitochondrial oxidative phosphorylation system, we grew cybrids in a glucose-free medium containing galactose; under these conditions, the rate of glycolysis is markedly reduced and cells are forced to rely on oxidative phosphorylation for ATP production [33]. No significant differences were found in LHON cell viability compared to controls (Fig 5A) or between different cell clones (S3 Fig). Assessment of CI redox activity displayed a non-significant reduction in LHON cells (Fig 5B), indicating that the combinations of variants did not affect the CI oxidoreductase function. However, the basal and the FCCP-stimulated oxygen consumption rate (OCR) of LHON cells were significantly reduced (Fig 5C). LHON cells also displayed a metabolic shift toward glycolysis, since they showed a higher ECAR and a lower OCR when compared to controls (Fig 5D). Consistently, the CS normalized ATP synthesis, driven by CI substrates (malate and glutamate), was significantly reduced in LHON cells, whereas ATP synthesis was normal when driven by CII substrates (succinate) (Fig 5E). The OCR analysis of a further cybrids cell line carrying the m.10680G>A/*MT-ND4L* variant in isolation, also displayed a defective respiration with a similar magnitude as LHON cells (Fig 5C and S4 Fig).

Although LHON mutations have been reported to exert their pathogenic role by increasing oxidative stress [4,5], we failed to reveal any difference between LHON and control cells in terms of superoxide anion and hydrogen peroxide production (S5 Fig). Overall, these data indicate that combinations of polymorphic variants in mtDNA-encoded CI genes induce a mild isolated CI defect, which was also detected in cells carrying the m.10680G>A/*MT-ND4L* variant in isolation.

### Modeling of the identified variants on the ovine Complex I crystal structure

In order to define how such peculiar combinations of variants lead to a mild Complex I defect, we took advantage of the recently released crystallographic structure of mammalian enzyme [34]. We analyzed the position of amino acids affected by the polymorphic variants, namely m.14258G>A/*MT-ND6*, m.14582A>G/*MT-ND6*, m.10680G>A/*MT-ND4L* and m.12033A>G/*MT-ND4*.

**Fig 6:**
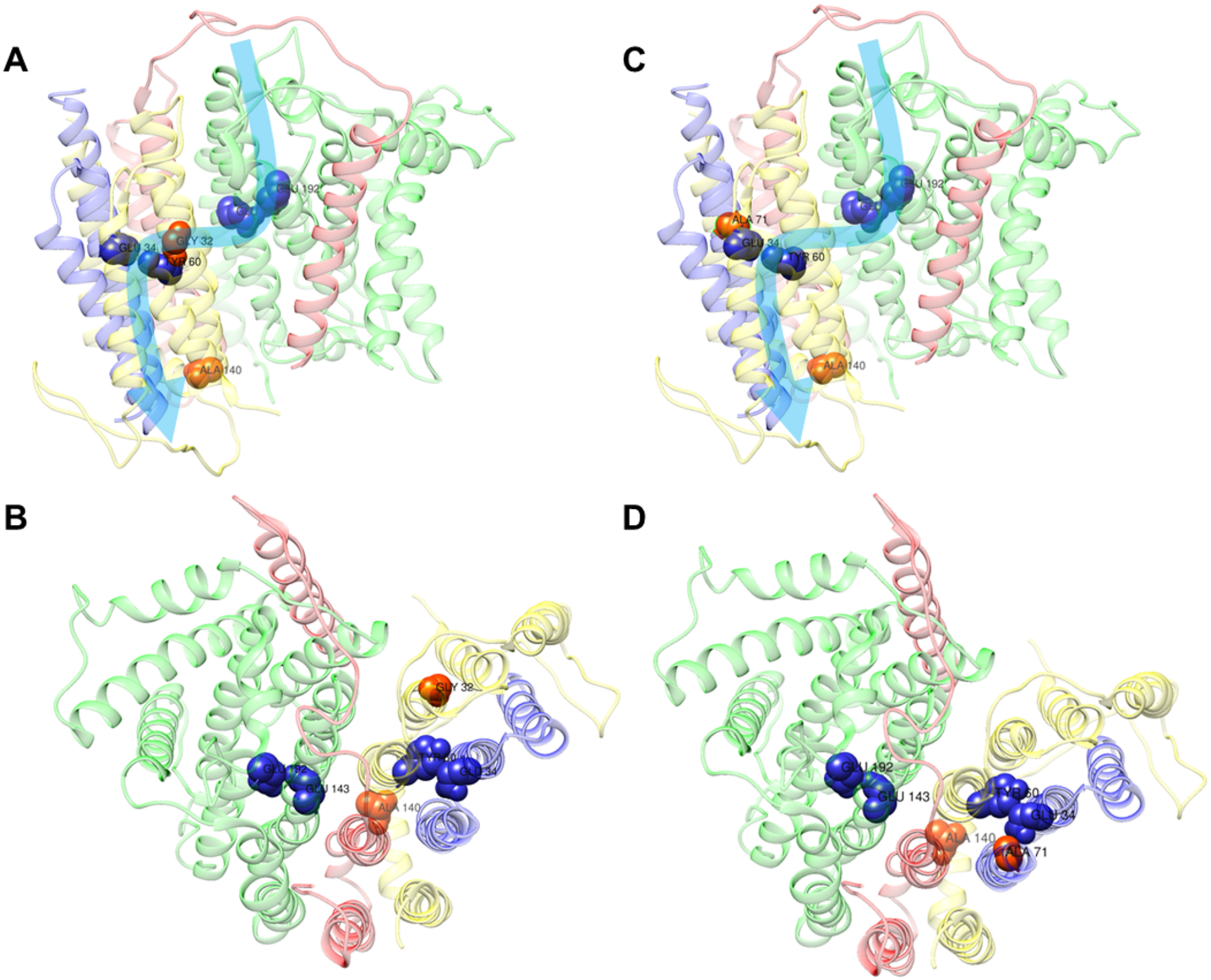
Complex I model. Localization of polymorphic variants on the cryo-EM structure of the ovine complex I, [34] using the UCSF Chimera software. The ovine amino acids Ala140 (corresponding to human p.P139L, m.14258G>A/*MT-ND6*), Gly32 (corresponding to human p.V31A, m.14582A>G/*MT-ND6*) and Ala71 (corresponding to human p.A71T, m.10680G>A/*MT-ND4L*) are shown as red-labelled spheres; whereas residues Glu143/ND1, Glu192/ND1, Glu34/ND4L, Tyr60/ND6, the key residues for the E-channel (near Q site), are shown as blue-labelled spheres. The structures of ND1, ND4L, ND6 and ND3 subunits are shown as ribbons, in green, blue, yellow and red, respectively. The combination of variants in Family 1 (**A-B**) and Family 2 (**C-D**) are displayed as front (**A-C**) and upper (**B-D**) views. Light blue arrows indicate the proposed proton translocation pathway [34].

The variant m.14258A>G/*MT-ND6* shared by Families 1 and 2 induces the P139L amino acid change in humans, which corresponds to A140 in ovine CI. Such amino acid is located in the transversal α-helix 5 of ND6. The variant m.14582A>G/*MT-ND6* found in Family 1 generates the amino acid substitution p.V31A in humans and corresponds to G32 in the ovine complex, affecting the transmembrane α-helix 2 (TM2) of ND6. The m.10680G>A/*MT-ND4L* variant harbored by Family 2 affects the amino acid A71 of ND4L both in human and ovine CI. This amino acid lies in TM3 of the ND4L subunit. Lastly, the m.12033A>G/*MT-ND4* induces the amino acid substitution p.N425S in the loop between TM13 and TM14 of ND4, which faces the mitochondrial matrix. Interestingly, with the only exception of the latter amino acid change, all the other variants affect positions around the putative E-channel of CI [35], suggesting that the mild functional defect found in these patients may arise from an altered proton pumping caused by the two peculiar mtDNA combinations of variants (Fig 6).

## Discussion

The current study provides genetic and functional evidence that specific and previously unreported combinations of missense mtDNA variants, which individually obey the definition of population polymorphisms, may exert a sufficient pathogenic potential for being causative of low-penetrance LHON. This, as confirmed by a few other cases retrieved from the literature, now firmly establishes that LHON is a disease that may be determined by a very mild respiratory chain dysfunction, possibly close to the boundary between functional and pathological variability, which depends on the mitogenome sequence variation [36,37], and is highly modulated by environmental [38] and nuclear DNA factors [39]. This scenario was best demonstrated by the cluster of Families 1a, 1b and 1c, which belong to the same maternal lineage and carry the previously unreported combination of m.14258G>A/*MT-ND6* and m.14582A>G/*MT-ND6* variants on a K1a haplogroup background (S5 Table). On the clinical ground, it is worth to notice that all affected members (*n* = 14) of this very large family are males, indicating that most likely the combination of missense mutations on a K1 mitogenome characterizing this family is not sufficient to reach and trespass the threshold for LHON in females. Furthermore, general penetrance of these 14 affected members was 13% over the total number of individuals on the maternal line, and 25% over the total number of males. This latter percentage is well below the usual quote of average 50% penetrance in males reported in literature [4,5].

It is also interesting to note that the mitogenomes of both Families 1 and 2 had in common m.14258G>A/*MT-ND6*, a mutation previously not recognized as associated with LHON. By screening the entire cohort of LHON families diagnosed in our Institute, we found this variant also in two LHON pedigrees carrying one of the three common LHON primary mutations (S1 and S2 Figs). The other variant m.10680G>A/*MT-ND4L* found in Family 2 has been either found alone in pedigrees segregating cases of LHON on the maternal line [31,32], or in association with known LHON primary mutations [26]. Moreover, the mtDNA sequence variants of these previously reported cases of Chinese ancestry are found in combination with other missense changes in CI subunits genes, in particular in the ND1 subunit (m.3644T>C/*MT-ND1*; m.3745G>A/*MT-ND1*; m.3548T>C/*MT-ND1*) or in the ND6 subunit (m.14484T>C/*MT-ND6*), which are closely assembled with ND4L according to the CI structure (S6 Fig, S6 Table) [34]. Thus, at least the m.14258G>A/*MT-ND6* and m.10680G>A/*MT-ND4L* variants have been recurrently associated with LHON, either in combination with other polymorphic variants or associated with other primary mutations. However, both variants alone are reported, at very low frequencies, in the general population excluding LHON cases (respectively 20 and 14 mitogenomes, out of 31,787), thus with an extremely low possibility of co-occurrence by chance, and consequently further remarking their non-pathogenicity when isolated (S5 and S6 Tables).

To validate on the functional ground the pathogenic role of these two combinations of variants, the only recognized method is to demonstrate a biochemical defect in the cybrid cell model, where only the patient-derived mtDNA is transferred. We observed that ATP synthesis driven by CI substrates and respiration as measured by OCR were significantly defective in cybrids harboring the mitogenomes of Families 1 and 2 compared to haplogroup-matched controls. Intriguingly, a similar defect in respiration was also found in a further cybrid cell line carrying the m.10680G>A/*MT-ND4L* missense variant in isolation on a I1b haplogroup, supporting its contributory role to the pathogenic potential of the Family 2 combination of variants. Unfortunately, not having available cybrids carrying the m.14258G>A/*MT-ND6* variant in isolation, we could not asses its real contribution to the respiratory defect of both Families 1 and 2 combinations of variants. However, in Family 1 the co-occurrence of the m.14582A>G/*MT-ND6* variant ultimately results in defective respiration of similar magnitude as in Family 2 and as in cybrids with the isolated m.10680G>A/*MT-ND4L* variant. Remarkably, similar to the other LHON primary mutations with the exception of m.3460G>A/*MT-ND1*, the CI specific activity was not reduced in cybrids carrying the two combinations of variants [40,41]. Thus, even if other reports proposed that combinations of different variants might exert the equivalent pathogenic role of the single primary LHON mutation [31,32], we here provide for the first time experimental evidence of the dysfunction from a functional point of view. Interestingly, the now available structure of CI revealed that the large majority of these variants, those found in the Italian Families 1 and 2 as well as those reported in Chinese families [31,32], are located in close proximity to the predicted E-channel for proton translocation (Fig 6, S6 Fig), contributed by all three ND6, ND4L and ND1 subunits. Genetic variation along this pathway may alter the efficiency of proton translocation, ultimately affecting the energy conserving function of CI.

Our study has profound implications for the diagnosis of LHON, and, more in general, for the assessment of pathogenicity of mtDNA variants. In the case of the three branches of Family 1, we performed complete mtDNA sequencing because there was a clear evidence of maternal recurrence of a phenotype undistinguishable from classic LHON despite the absence of the three common LHON mutations. However, also the sequencing of the entire mitogenome in our entire cohort of Italian LHON families revealed the presence of multiple variants potentially relevant for LHON pathogenesis, beside the known primary LHON mutations. Therefore, we propose complete mitogenome sequencing as the gold standard for LHON diagnosis, to disclose possible unique combinations of variants, or double/triple mutants. In brief, the definition of a pathogenic mtDNA mutation becomes blurrier than ever, and only the accurate consideration of population-dependent mtDNA structure, combined with functional analyses using the cybrid cell model, may lead to its final validation. A good example for such a scenario is the m.3394T>C/*MT-ND1*, which might act as an adaptive variant selected for high altitudes in Tibet, while exerting a pathogenic effect on other mtDNA backgrounds and predisposing to LHON in China [27]. Closely similar, other two adaptive variants for high altitude in Tibet, i.e. m.3745G>A/*MT-ND1* and m.4216T>C/*MT-ND1*, were also implicated in LHON [42]. The m.3745G>A/*MT-ND1* was in fact found in combination with m.10680G>A/*MT-ND4L* in a Chinese LHON Family [26], whereas the m.4216T>C/*MT-ND1* variant is at the shared root of the Western Eurasian haplogroups J and T, both possibly affecting the E-channel for proton pumping (S6 Fig).

In conclusion, this study highlights the complexities of mtDNA sequence variability, introducing a perspective that will change the approach for assigning the pathogenic role to peculiar combinations of mtDNA variants, and modifying the criteria [3] for diagnostics in mitochondrial human diseases.

## Materials and methods

### Skeletal muscle investigation

Tibialis anterior muscle biopsy was carried out after informed consent from patients. Routine histological and histoenzymatic analyses, including cytochrome c oxidase (COX) and succinic dehydrogenase (SDH) activity staining, were performed [43]. Respiratory chain complexes and CS activities were determined on skeletal muscle homogenates as previously reported with minor modifications [44]. Skeletal muscle biopsy was also processed for transmission electron microscopy (TEM) using standard procedures.

### mtDNA sequencing and copy number quantification

Total DNA was extracted by standard methods from blood cells, urinary sediment epithelium and skeletal muscle after informed consent and approval of the internal review board at University of Bologna. Direct sequence analysis of the entire mtDNA molecule was performed on total DNA extracted from skeletal muscle, by Sanger [45] or Next Generation Sequencing (NGS) methods. For the NGS approach, briefly, two long PCR amplicons (9.1 kb and 11.2 kb) [46] were amplified using Q5 High-Fidelity DNA Polymerase (New England Biolabs, UK), purified by Agencourt AMPure XP (Beckman Coulter Life Sciences, Italy). The library was constructed by Nextera XT DNA Library Preparation Kit (Illumina, San Diego, CA) and sequenced on MiSeq System (Illumina, San Diego, CA), using the 600-cycle reagent kit.

All the mutations are relative to the revised Cambridge Reference Sequence (rCRS, NC_012920). The complete mtDNA sequence of the three maternally linked probands of Family 1 (GenBank: KC878720) as well as that from the proband of Family 2 (GenBank: MF039863) have been deposited. All variants of interest were confirmed in all their available maternal relatives by restriction fragment length polymorphism (RFLP) analysis (primers and conditions are available upon request). Mitochondrial DNA copy number was evaluated by qRT-PCR, as previously reported [28]. Population frequencies of missense mutations and the mtDNA backgrounds on which they were observed were recovered from two public databases, Mitomap (http://www.mitomap.org) and HmtDB (http://www.hmtdb.uniba.it) [47,48]. Haplogroup affiliations of mitogenomes were assigned according to PhyloTree (www.phylotree.org) [49].

### Protein conservation analysis and homology modelling

Protein conservation analysis and pathogenicity prediction were carried out applying a previously detailed *in silico* protocol [21] and MitImpact 2.7 (mitimpact.css-mendel.it) [35]. Positioning of amino acid changes on the 3D CI structure was performed using UCSF Chimera 1.11.2 (www.cgl.ucsf.edu/chimera/) on the entire ovine respiratory Complex I (PDB file 5LNK) [34].

### Generation and maintenance of cybrids

Cybrid cell lines were generated from patient’s skin fibroblasts (individuals IV:1 from Family 1a; V:6 from Family 1b; and IV:2 from Family 2) and 143B.Tk^-^ cells, as previously described [50]. A further cybrid cell line was generated from fibroblasts carrying the m.10680G>A/*MT-ND4L* in isolation (haplogroup I1b), identified after screening our entire fibroblast biobank from patients without mtDNA-based neurological disorders. Cybrids from control fibroblasts were previously generated (GeneBank MF591562, EU915473, MF591564) and used in this study after the closest mtDNA haplogroup matching with LHON patients cybrids (N1b1a, K1a2a and H1, respectively).

Cybrids were grown in complete medium Dulbecco’s modified Eagle medium (DMEM) supplemented with 10% fetal calf serum (South America source from Gibco, Life Technologies, Italy), 2 mM L-glutamine, 100 U/ml penicillin, 100 μg/ml streptomycin, in an incubator with a humidified atmosphere of 5% CO_2_ at 37 °C. All the experiments were performed using haplogroup-matched wild type controls.

### Cell viability assessment

For viability experiments, cells (4x10^4^ cells/cm^2^) were seeded in 24 well plates and incubated for different times in complete medium or in glucose-free DMEM supplemented with 5 mmol/L galactose, 5 mmol/L Na-pyruvate and 5% FBS (DMEM-galactose). Viability was determined using the colorimetric sulforhodamine B (SRB) assay [51], by measuring the SRB absorbance at 570 nm with a VICTOR^3^ Multilabel Plate Counter (PerkinElmer Life and Analytical Sciences, Zaventem, Belgium).

### Mitochondria preparation and Complex I activity

Isolation of mitochondrial-enriched fraction and assessment of Complex I activity were carried out as previously described [52]. Rotenone sensitive specific Complex I activity was normalized on protein content and CS activity [44].

### Oxygen consumption rate

Oxygen consumption rate (OCR) and extracellular acidification rate (ECAR) in adherent cells were measured with an XFe^24^ Extracellular Flux Analyzer (Seahorse Bioscience, Billerica, MA, USA), as previously described [53]. OCR and ECAR were measured under basal conditions and after the sequential addition of 1μM oligomycin, 0.2μM FCCP (carbonylcyanide-p-trifluoromethoxyphenyl hydrazone, Sigma-Aldrich, Milan, Italy), 1μM rotenone and 1μM antimycin A. Data were normalized on SRB absorbance values and on non-mitochondrial residual OCR, after antimycin injection, and expressed as percentage.

### ATP synthesis

The rate of mitochondrial ATP synthesis was measured in digitonin-permeabilized cybrids using the previously described luciferin/luciferase assay, with minor modifications [54]. Rates were normalized to protein content and CS activity [44].

### Reactive oxygen species (ROS) assessment

Quantification of mitochondrial superoxide and H_2_O_2_ levels were performed by flow cytometry or fluorescent microscopy using H_2_DCFDA and MitoSox fluorescent dies (Life Technologies, Milan, Italy), as previously detailed [53].

### Statistical analyses

Statistical significance was defined as p-value≤0.05 with Student’s t-test unless otherwise indicated.

## Supporting information

S1 Text Pedigrees and case reports. (DOCX)

S1 Table Ophthalmological data. (DOCX)

S2 Table Clinical data. (DOCX)

S3 Table Respiratory chain enzyme activity on skeletal muscle normalized for CS activity. (DOCX)

S4 Table Prediction tools (MitImpact 2.7) and conservation analysis. (DOCX)

S5 Table Mitogenome sequences carrying the m.14258G>A/*MT-ND6*, p.P139L, in common databases. (DOCX)

S6 Table Mitogenome sequences carrying the m.10680G>A/*MT-ND4L*, p.A71T, in common databases. (DOCX)

S1 Fig Family 3 with ophthalmologic and clinical features. (TIF)

S2 Fig Family 4 with ophthalmologic and clinical features. (TIFF)

S3 Fig Cell viability of individual cybrid clones of the three analyzed cell lines carrying combinations of polymorphic variants in CI genes. A. Cell viability in complete medium (25 mM glucose) for different times (0, 24, 48, 72h). Data are expressed as percentage of T0 (*n* = 3; mean ± SD). B. Cell viability in galactose (5 mM) medium for different times (0, 24, 48, 72h). Data are expressed as percentage of T0 (*n* = 3; mean ± SD).
(TIF)

S4 Fig Oxygen consumption rate (OCR) in cybrids carrying the individual polymorphic variant m.10680G>A/*MT-ND4L*. OCR traces as pmol O_2_/min, after the injection of 1μM oligomycin (O), 0.2μM FCCP (F), 1μM rotenone (R) and 1μM antimycin A (AA) (mean ± SD). Asterisks indicate statistical significance (*n* = 3; * p<0.05).
(TIF)

S5 Fig Production of superoxide anion and hydrogen peroxide in cybrid cell lines. **A**. Mitochondrial superoxide anion production determined by epifluorescence microscopy using MitoSOX fluorescent dye. Cells were visualized with a digital imaging system, using an inverted epifluorescence microscope (magnification x63/1.4 oil objective) at 580nm. Images are representative of 3 different experiments. **B**. Hydrogen peroxide levels were measured using H_2_DCFDA by flow cytometry, as described in materials and methods. Data are mean ± SD (*n* = 3).
(TIFF)

S6 Fig Modeling of combinations of mtDNA variants from three LHON Chinese families and single mtDNA variants, adaptive for high altitude in Tibet, on the ovine Complex I structure. Positioning of the combinations of variants in three LHON Chinese families (**A-B**) [26,31,32] and the adaptive variants for high altitude in Tibet (**C-D**) [27,42] on structure of ovine CI obtained by cryo-EM [34], using the UCSF Chimera software. In panels **A** and **B**, the ovine amino acid Ala71 (corresponding to human p.A71T, m.10680G>A/*MT-ND4L*) is shown as red labelled sphere, and this variant is associated in each family with Met65 (corresponding to human p.M64V, m.14484T>C /*MT-ND6*), Ala147 (corresponding to human p.A147T, m.3745G>A/*MT-ND1*), Leu81 (corresponding to human p.I81T, m.3548T>C/*MT-ND1*) or Val113 (corresponding to human p.V113A, m.3644T>C/*MT-ND1*), shown as green labelled spheres. In panels **C** and **D**, the ovine amino acids Ala147 (corresponding to p.A147T, m.3745G>A/*MT-ND1*), His304 (corresponding to human p.Y139H, m.4216T>C/*MT-ND1*) and Tyr30 (corresponding to human p.Y30H, m.3394T>C/*MT-ND1*) are shown as green labelled spheres. In all panels, residues Glu143/ND1, Glu192/ND1, Glu34/ND4L, Tyr60/ND6, the key residues for the E-channel (near Q-site), are shown as blue labelled spheres [27]. The backbones of ND1, ND4L, ND6 and ND3 are shown as ribbons, in green, blue, yellow and red, respectively. The variants combination in LHON Chinese families (**A**-**B**) and adaptive variants for high altitude in Tibet (**C**-**D**) are displayed as front (**A**-**C**) and upper (**B**-**D**) views. Light blue arrows indicate the proposed proton translocation pathway.
(TIFF)
