# Supplementary material for: Peculiar combinations of individually non-pathogenic missense mitochondrial DNA variants cause low penetrance Leber’s hereditary optic neuropathy
Source: PLoS Genet. 2018 Feb 14;14(2):e1007210. doi: 10.1371/journal.pgen.1007210 (PMC5828459; doi:10.1371/journal.pgen.1007210)
Supplement: S1 Text — (DOCX) [file pgen.1007210.s001.docx]

**S1 Text. Pedigrees and case reports**

**Family 1a.** Family 1a is a multigenerational maternal lineage (Fig 1) originating from the region Campania in southern Italy, with three similarly affected maternally-related males including the proband (IV:1), one first cousin (IV:4) and one distant uncle (III:14). Available ophthalmological data for all affected individuals are reported in S1 Table, whereas further clinical information is in S2 Table.

We here briefly summarize the clinical history of the proband (IV:1). This 29-year-old patient presented an acute visual loss in OD at 16 years of age, followed after 20 days by visual loss in OS. His clinical features were typical of LHON, but genetic analysis was negative for the three common LHON mutations. Idebenone therapy was started at 17 years of age (270 mg/die) without benefit at follow-up visits.

We first evaluated this patient at 18 years of age. Neurological examination was normal except for postural tremor and sporadic parcellar myoclonic jerks at upper limbs. Lactic acid after standardized effort was within normal limits [1]. Visual evoked potentials (flash, VEPs) showed the absence of cortical responses in OD and delayed latencies in OS. EKG showed a mild delay in right conduction time. At 21 years of age, a follow-up ophthalmologic evaluation showed that visual acuity (VA) was 1/20 bilaterally with diffuse optic atrophy (Fig 2A). At the last ophthalmologic evaluation (29 years), VA was 0.032 bilaterally.

**Family 1b.** Family 1b is also a multigenerational maternal lineage (Fig 1) originating, as Family 1a, from the region Campania. The maternal ancestors of Family 1a (I:2) and b (I:2) carried the same family name, and the two families live in two different but close villages from the same small geographical area. Family history is relevant for five males similarly affected by visual loss, including the proband (V:6), his brother (V:5), two maternal uncles (IV:4; IV:6) and the brother of the maternal grandmother (III-5), who also had a diagnosis of atypical parkinsonism. Available ophthalmological and clinical data for the proband and his brother are summarized in supplementary Tables 1 and 2.

The proband (V:6), a 28-year-old male, presented with acute loss of vision in OS at 18 years of age, when we first evaluated him. Despite he was negative for the three LHON common mutations, the maternal recurrence of other optic neuropathy cases and the clinical features indistinguishable from LHON prompted to start idebenone therapy at 900 mg/day, five months after onset. The dosage was reduced to 270 mg/day one year later and further reduced at 180 mg/day three years later. The patient subjectively perceived improvement of vision in OS one year after disease onset and six months after therapy onset, with documented recovery of visual acuity three years after onset, also confirmed by visual fields and visual evoked potentials. The last ophthalmological evaluation revealed visual acuity OD 10/10 and OS 1/10 with small central scotoma in OS (Fig 2B). Interestingly, both his maternal uncles (IV:4; IV:6) were reported as suffering unilateral visual loss.

Relevant co-morbidities were, since childhood, migraine and panic attacks with anxiety treated with SSRI since 16 years of age. Furthermore, since age 19 he also complained of jerks at lower limbs, appearing in relaxed wakefulness and persistent in sleep. Polysomnographic recording disclosed the presence of periodic limb movements. Polygraphic recording showed a normal EEG activity with myoclonic jerks without a cortical correlate at back-average analysis. Approximately from the same age, he also complained of myalgia and cramps. Skin biopsy revealed the presence of a small fiber neuropathy.

The last neurological examination, at 28 years of age, was significant for the presence of postural tremor at upper limbs, parcellar and segmental myoclonic jerks at upper limbs both spontaneous and evoked, brisk deep tendon reflexes (right>left). Lactic acid evaluation showed pathologic values after exercise (37 mg/dl, normal range 5-18 mg/dl). Somatosensory and motor evoked potentials were normal, as well as electromyography. Brain magnetic resonance and cerebral (^1^H) and muscle (^31^P) MR spectroscopy were also normal.

**Family 1c.** Family 1c is a third large maternal lineage (Fig 1), as Families 1a and 1b, originating from the region Campania, but from a town relatively distant from the small geographical area where both Families 1a and 1b live. Family history is relevant for six males similarly affected by visual loss including the proband (IV:1), his maternal uncle (III:7) and granduncle (II:3), and three maternally related first cousins of the maternal uncle (III:15; III:16; III:17). Available ophthalmological and clinical data from the proband and his maternal uncle are summarized in supplementary Tables 1 and 2.

The proband (IV:1), now 25-year-old, lost vision bilaterally when he was 16 with the typical hallmarks of LHON. Genetic investigation was negative for the three common LHON mutations. We observed this patient after five years from disease onset. His visual acuity was 7-8/10 bilaterally with central scotoma at visual fields, having experienced a spontaneous recovery of visual acuity from a nadir of 1/10 bilaterally. A therapy with idebenone (405 mg/die) was started. Neurological exam was unremarkable and lactic acid after effort was slightly elevated (28 mg/dl, normal range 5-22 mg/dl).

**Family 2.** Family 2 is a multigenerational maternal lineage with a single affected patient (IV:2) (Fig 1) originating from the region Emilia-Romagna in northern Italy. This 22-year-old male presented acute loss of vision in OD at 16 years of age after head trauma, being hit violently by a soccer ball. After six months, when we observed for the first time the patient (at 17 years of age), he reported reduction of visual acuity in OS, which worsened in the following months. He was suspected having LHON, but the genetic investigation was negative for the three common mutations. However, idebenone therapy was started at 675 mg/day and improvement of visual function was subjectively reported two months later.

Neurological examination was unremarkable. Lactic acid evaluation showed abnormally elevated values after exercise (38.6 mg/dl, normal range 5-22 mg/dl). Brain magnetic resonance and ^1^H-MRS spectroscopy were normal. EKG showed a mild right conduction delay. VEPs showed delayed conduction time bilaterally (R>L). Further clinical data are summarized in supplementary Tables 1 and 2.

The patient progressively improved his visual function, as documented by visual acuity and visual field assessments and, at the last evaluation, he had 10/10 in both eyes (Fig 2C).

**Supplementary References**

1. Montagna P, Plazzi G, Cortelli P, Carelli V, Lugaresi E, Barboni P, et al. Abnormal lactate after effort in healthy carriers of Leber’s hereditary optic neuropathy. J Neurol Neurosurg Psychiatry. 1995;58: 640–641.

2. Carelli V, Barboni P, Zacchini A, Mancini R, Monari L, Cevoli S, et al. Leber’s Hereditary Optic Neuropathy (LHON) with 14484/ND6 mutation in a North African patient. J Neurol Sci. 1998;160: 183–188.

3. Torroni A, Carelli V, Petrozzi M, Terracina M, Barboni P, Malpassi P, et al. Detection of the mtDNA 14484 mutation on an African-specific haplotype: implications about its role in causing Leber hereditary optic neuropathy. Am J Hum Genet. 1996;59: 248–252.
