# Supplementary material for: Peculiar combinations of individually non-pathogenic missense mitochondrial DNA variants cause low penetrance Leber’s hereditary optic neuropathy
Source: PLoS Genet. 2018 Feb 14;14(2):e1007210. doi: 10.1371/journal.pgen.1007210 (PMC5828459; doi:10.1371/journal.pgen.1007210)
Supplement: S1 Table — (DOCX) [file pgen.1007210.s002.docx]

**S1Table.** Ophthalmological data

| **Family** | **Subject** | **Age** | **Age at onset** | **Recovery** | **Start of therapy** | **Visual acuity** | **Visual fields**  **(Mean deviation)** | **Optical Coherence Tomography** | **Fundus Oculi**  **(ONH)** |
| --- | --- | --- | --- | --- | --- | --- | --- | --- | --- |
| 1a | IV:1 | 29 | 16 ys | No | 17 ys | OO 3/100 | Diffuse defect bilaterally  OD -27.72 OS -27.33 | Diffuse loss of fibers bilaterally  with nasal sparing | Diffuse pallor bilaterally |
| 1a | IV:4 | 13 | 9 ys | Yes | 10 ys | OO 1/10 | Bilateral central scotoma  OD -4.15 OS -5.20 | OD: Diffuse loss of fibers with nasal sparing;  OS: Loss of fibers in supero-temporal sectors | Temporal pallor bilaterally |
| 1a | III:14 | 44 | 40 ys | No | 42 ys | OD 1/10  OS 1.6/10 | Bilateral central scotoma  OD -9.05 OS -28.43 | OD: Diffuse loss of fibers  OS: temporal loss of fibers | Mild temporal pallor |
| 1b | V:6 | 28 | 18 ys (OS) | Yes  (21 ys) | 18 ys | OD 10/10  OS 1/10 | OD: normal  OS: small central scotoma  (-2.66) | OD: normal  OS: temporal loss of fibers | OD: normal  OS: temporal pallor |
| 1b | V:5 | 41 | 27 ys | Yes  (31 ys) | 32 ys | OO 1/20 | n.a. | n.a. | n.a. |
| 1c | III:7 | 57 | 16 ys | Yes | 52 ys | OO 1/20 | Diffuse defect bilaterally  OD -17.68 OS -17.84 | Diffuse loss of fibers bilaterally | Diffuse pallor bilaterally |
| 1c | IV:1 | 25 | 16 ys | Yes | 21 ys | OO  7-8/10 | Bilateral central scotoma  OD -6.07  OS -6.06 | Temporal and inferior loss of fibers bilaterally (OS>OD) | Temporal pallor bilaterally |
| 2 | IV:2 | 22 | 16 ys  (after a head trauma) | Yes  (april 2011,  17 ys) | 17 ys | OO 10/10 | Bilateral central scotoma  OD -5.36  OS -8.21 | Diffuse loss of fibers bilaterally with nasal sparing | Diffuse pallor bilaterally |

OS=left eye; OD=right eye; OO=both eyes; n.a.=not available; ONH=optic nerve head
