# Supplementary material for: Peculiar combinations of individually non-pathogenic missense mitochondrial DNA variants cause low penetrance Leber’s hereditary optic neuropathy
Source: PLoS Genet. 2018 Feb 14;14(2):e1007210. doi: 10.1371/journal.pgen.1007210 (PMC5828459; doi:10.1371/journal.pgen.1007210)
Supplement: S2 Table — (DOCX) [file pgen.1007210.s003.docx]

**S2 Table.** Clinical data

| **Family** | **Subject** | **Age** | **Neurological exam** | **Lactic acid_*_** | **Muscle biopsy** | **EMG** | **Brain**  **H^1-^MRS** | **Audiometry** | **Other** |
| --- | --- | --- | --- | --- | --- | --- | --- | --- | --- |
| 1a | IV:1 | 29 | Postural Tremor  Myoclonic jerks | 15.5-13.9-22.3-17 | Fiber size variability, SDH increase | n.a. | n.a. | n.a. |  |
| 1a | IV:4 | 13 | n.a. | n.a. | n.a. | n.a. | n.a. | n.a. | Brain MRI: normal |
| 1a | III:14 | 44 | Hypopallestesia at lower limbs | n.a. | n.a. | n.a. | n.a. | n.a. | Ischemic cardiopathy  Brain MRI: normal |
| 1b | V:6 | 28 | Postural Tremor  Myoclonic jerks | 13-12-37-21 | Fiber size variability, SDH increase | normal | normal | n.a. | PLMD since 19 years; panic attacks and anxiety disorder; migraine since childhood; small fibers neuropathy |
| 1b | V:5 | 41 | Brisk deep tendon reflexes | 13.7-13-14.3-13 | n.a. | n.a. | n.a. | n.a. |  |
| 1c | III:7 | 57 | Weak deep tendon reflexes | n.a. | n.a. | n.a. | n.a. | Mild sensorineural deafness | Diabetes since 40 yrs  Brain MRI normal |
| 1c | IV:1 | 25 | normal | 14-14-28-15 | n.a. | n.a. | n.a. | n.a. | Brain MRI normal |
| 2 | IV:2 | 22 | normal | 8.4-8.2-38.6-17 | Fiber size variability, SDH increase | normal | normal | n.a. |  |

n.a.=not available; EMG= electromyography; MRS= magnetic resonance spectroscopy; MRI= magnetic resonance imaging; PLM=periodic limb movements.

*Lactic acid normal values 5-22 mg/dl
