# Supplementary material for: Peculiar combinations of individually non-pathogenic missense mitochondrial DNA variants cause low penetrance Leber’s hereditary optic neuropathy
Source: PLoS Genet. 2018 Feb 14;14(2):e1007210. doi: 10.1371/journal.pgen.1007210 (PMC5828459; doi:10.1371/journal.pgen.1007210)
Supplement: S3 Table — (DOCX) [file pgen.1007210.s004.docx]

**S3 Table.** Respiratory chain enzyme activity on skeletal muscle normalized for CS activity

|  | **CI** | **CII+III** | **CIII** | **CIV** |
| --- | --- | --- | --- | --- |
| Controls (mean ± SD) | 31.7±20.0 | 20.3±18.5 | 197.94±119.8 | 54.6±23.6 |
| Family 1a IV:1 | 48.10 | 29.32 | 202.63 | 85.23 |
| Family 2 IV:2 | 31.93 | 35.83 | 281.37 | 68.25 |
