# Supplementary material for: Peculiar combinations of individually non-pathogenic missense mitochondrial DNA variants cause low penetrance Leber’s hereditary optic neuropathy
Source: PLoS Genet. 2018 Feb 14;14(2):e1007210. doi: 10.1371/journal.pgen.1007210 (PMC5828459; doi:10.1371/journal.pgen.1007210)
Supplement: S4 Table — (DOCX) [file pgen.1007210.s005.docx]

**S4 Table.** Prediction tools (MitImpact 2.7) and conservation analysis

| Family | 1,2 | 1 | 2 | 2 |
| --- | --- | --- | --- | --- |
| Mutation | m.14258G>A | m.14582 A>G | m.10680 G>A | m.12033 A>G |
| MitImpact_id | MI.23322 | MI.23997 | MI.16139 | MI.18998 |
| Gene_symbol | *MT-ND6* | *MT-ND6* | *MT-ND4L* | *MT-ND4* |
| AA_change | p.P139L | p.V31A | p.A71T | p.N425S |
| AA_ref | P | V | A | N |
| AA_alt | L | A | T | S |
| Codon_substitution | cCt/cTt | gTa/gCa | Gca/Aca | aAc/aGc |
| PhyloP conservation index | -1,44 | -12,89 | 0,06 | -0,66 |
| PhastCons conservation index | 0 | 0 | 0,94 | 0 |
| SiteVar conservation index | 0 | 0,03 | 0,01 | 0 |
| PolyPhen2 | benign | benign | benign | benign |
| SIFT | neutral | neutral | neutral | neutral |
| FatHmm | neutral | neutral | neutral | neutral |
| FatHmm Weighted | deleterious | neutral | neutral | neutral |
| PROVEAN | neutral | neutral | deleterious | neutral |
| MutationAssessor | neutral_impact | neutral_impact | high_impact | low_impact |
| EFIN SP | neutral | neutral | neutral | neutral |
| EFIN HD | neutral | neutral | neutral | neutral |
| CADD | deleterious | neutral | neutral | neutral |
| PANTHER | neutral | neutral | disease | neutral |
| PhD-SNP | neutral | neutral | disease | neutral |
| SNAP | neutral | neutral | disease | neutral |
| Meta-SNP | neutral | neutral | disease | neutral |
| CAROL | neutral | neutral | neutral | neutral |
| Condel | deleterious | deleterious | deleterious | deleterious |
| COVEC_WMV | neutral | neutral | neutral | neutral |
| MtoolBox | neutral | neutral | neutral | deleterious |
| PolyPhen2 transformed | medium_impact | medium_impact | medium_impact | medium_impact |
| SIFT transformed | medium_impact | medium_impact | medium_impact | medium_impact |
| MutationAssessor transformed | medium_impact | medium_impact | high_impact | medium_impact |
| APOGEE_boost | N | N | N | N |
| APOGEE_boost_mean_prob | 0,31 | 0,26 | 0,42 | 0,44 |
| SNPDryad_score | 0,72 | 0,11 | 0,02 | 0,01 |
| MutationTaster | NA | NA | disease_causing | polymorphism |
| MutationTaster_score | NA | NA | 0,97 | 1 |
| dbSNP_v149 | rs202227543 | rs41354845 | NA | NA |
| Mitomap_Dec2016_Status | NA | NA | Reported-possibly synergistic | NA |
| Mitomap_Dec2016_Disease | NA | NA | LHON | NA |
| GB | 16/32,059 | 179/32,059 | 14/32,059 | 5/32,059 |
| Frequency | 19/31,735 | 188/31,735 | 14/31,735 | 6/31,735 |
| **Conservation** | | | | |
| Eukaryota | F 22% | G 49% | A 86% | N 42% |
| Vertebrata | F 32% | G 69% | A 97% | N 54% |
| Mammalia | A 37% | G 46% | A 97% | N 70% |
| Local | 63% | 85% | 87% | 77% |
| Global | 71% | 71% | 80% | 83% |
