# Supplementary material for: Peculiar combinations of individually non-pathogenic missense mitochondrial DNA variants cause low penetrance Leber’s hereditary optic neuropathy
Source: PLoS Genet. 2018 Feb 14;14(2):e1007210. doi: 10.1371/journal.pgen.1007210 (PMC5828459; doi:10.1371/journal.pgen.1007210)
Supplement: S5 Table — (DOCX) [file pgen.1007210.s006.docx]

**S5 Table.** Mitogenome sequences carrying the m.14258G>A/*MT-ND6*, p.P139L in common databases (a total of 31,787 mitogenomes). The Family 2 proband is also included.

| **Genbank-HGDP-1000 GP ID** | **HmtDB Genome Identifier** | **Haplo-group** | **Private Variants** | | | **Haplogroup Diagnostic Missense Variants** | **Number of Mutational Events**  **m.14258G>A** | **Phenotype** |
| --- | --- | --- | --- | --- | --- | --- | --- | --- |
|  |  |  | **Synonymous** | **Non-Coding (for protein)** | **Missense** |  |  |  |
| MF039863 | Family 2 proband | H5b | m.10248T>C/MT-ND3 | *16309A>C/MT-HV1* | m.9966G>A*/MT-CO3* (V254I), m.10680G>A/*MT-ND4L* (A71T), m.12033A>G/*MT-ND4* (N425S), 14258G>A/*MT-ND6* (P139L)^e^ |  | 1 | LHON |
|  | PA_EU_XX_0017 | H5b | 8251G>A/*MT-CO2* | 16309A>G/*MT-HV1* | 9966G>A/*MT-CO3* (V254I) 12033A>G/*MT-ND4* (N425S) 14258G>A/*MT-ND6* (P139L)^e^ |  |  | Glioblastoma |
| HG00589 | AS_CN_0419 | F1a1 | 8697G>A/*MT-ATP6* | 203G>C/*MT-HV2* 1709G>A/*MT-RNR2* 16399A>G/*MT-HV1* | 14258G>A/*MT-ND6* (P139L) | 9053G>A/*MT-ATP6* (S176N) 10609 T>C/*MT-ND4L* (M47T) 12406 G>A/*MT-ND5* (V24I) 13759 G>A/*MT-ND5* (A475T) 13928 G>C/*MT-ND5* (S531T) | 1 | Normal |
| EF657644 | EU_XX_0538 | H1q3^a^ | 5237A>G/*MT-ND2* | 1009T>C/*MT-RNR1* | 9948G>A/*MT-CO3* (V248I) | 14258G>A/*MT-ND6* (P139L) | 1 | Normal |
| JX153975 | EU_DK_0876 | H1q3^a^ |  |  | 3511A>G/*MT-ND1* (T69A) | 14258G>A/*MT-ND6* (P139L) |  | Normal |
| KF161678 | EU_DK_1085 | H1q3^a^ |  |  | 3511A>G/*MT-ND1* (T69A) | 14258G>A/*MT-ND6* (P139L) |  | Normal |
| KF162479 | PA_EU_DK_0471 | H1q3^a^ |  |  | 3511A>G/*MT-ND1* (T69A) | 14258G>A/*MT-ND6* (P139L) |  | Diabetes |
| KM252740 | XX_XX_5717 | H1q3^a^ | 11266C>T/*MT-ND4* |  | 4084G>A/*MT-ND1* (V260D) | 14258G>A/*MT-ND6* (P139L) |  | Normal |
|  | PA_EU_XX_0019 | H1q3^a^ |  |  |  | 14258G>A/*MT-ND6* (P139L) |  | Glioblastoma |
| NA20811 | EU_IT_0707 | H1q3^a^ |  |  |  | 14258G>A/*MT-ND6* (P139L) |  | Normal |
| KP340158 | n.a. | HV2a2^b^ | 6563C>T/*MT-CO1* |  | 14258G>A/*MT-ND6* (P139L) |  | 1 | n.a. |
| KP340159 | n.a. | HV2a2^b^ | 6563C>T/ *MT-CO1* |  | 14258G>A/*MT-ND6* (P139L) |  |  | n.a. |
| KC878720 | EU_IT_0585 | K1a | 6137T>C/ *MT-CO1* 6329C>T/ *MT-CO1* 8994G>A/*MT-ATP6* 11038A>G/*MT-ND4* 15253A>G/*MT-CYB* |  | 14258G>A/*MT-ND6* (P139L) 14582A>G/*MT-ND6* (V31A) | 9055G>A/*MT-ATP6* (A177T) 14798T>C/*MT-CYB* (F18L) | 1 | Normal |
| KC533510 | PA_AF_SF_0059 | L0d2a1 | 10771A>G/*MT-ND4* |  | 5460G>A/*MT-ND2* (A331T) 13508C>T/*MT-ND5* (S391F) 14258G>A/*MT-ND6* (P139L) | 4025C>T/*MT-ND1* (T240M) 4225A>G/*MT-ND1* (M307V) 4232T>C/*MT-ND1* (I309T) 5442T>C/*MT-ND2* (F325L) | 1 | Pediatric patients |
| NA18868 | AF_NG_0078 | L2a1c1a1 |  | 2242T>C/*MT-RNR2* | 8584G>A/*MT-ATP6* (A20T) 14258G>A/*MT-ND6* (P139L) | 3308T>C/*MT-ND1* (M1T) 3338T>C/*MT-ND1* (V11A) 6663A>G/*MT-CO1* (I254V) 8584G>A/*MT-ATP6* (A20T) | 1 | Normal |
| KF451170/ HGDP00647 | XX_XX_6141 | U1b1 | 9374A>G/*MT-CO3* | 444A>G/*MT-DLOOP* 2352T>C/*MT-RNR2* | 14258G>A/*MT-ND6* (P139L) | 15110G>A/*MT-CYB* (A122T) | 1 | Normal |
| JN203207 | EU_PL_0054 | U3a1a1^c^ |  |  | 3808A>G/*MT-ND1* (T168A) | 10506A>G/*MT-ND4L* (T13A 13934C>T/*MT-ND5* (T533M) 14258G>A/*MT-ND6* (P139L) | 1 | Normal |
| JQ704950 | EU_IE_0218 | U3a1a1^c^ | 9656T>C/*MT-CO3* 14049C>T/*MT-ND5* |  |  | 10506A>G/*MT-ND4L* (T13A) 13934C>T/*MT-ND5* (T533M) 14258G>A/*MT-ND6* (P139L) |  | Normal |
| JX153017 | EU_IT_0472 | U3a1a1^c^ | 13785C>T/*MT-ND5* |  |  | 10506A>G/*MT-ND4L* (T13A) 13934C>T/*MT-ND5* (T533M) 14258G>A/*MT-ND6* (P139L) |  | Normal |
| HM156692 | PA_XX_XX_0032 | W3a1b^d^ | 8251G>A*/MT-CO2* | 146T>C/*MT-HV2* | 3350T>C/*MT-ND1* (I15T) 14258G>A/*MT-ND6* (P139L) | 3505A>G/*MT-ND1* (T67A) 5046G>A/*MT-ND2* (V193I) 15884G>C/*MT-CYB* (A380P) | 1 | Chronic Periodontitis |
| HM156696 | PA_XX_XX_0028 | W3a1b^d^ | 8251G>A/*MT-CO2* 8952T>C/*MT-CO2* 8994G>A/*MT-CO2* | 1243T>C/*MT-RNR1* | 3505A>G/*MT-ND1* (T67A) 5460G>A/*MT-ND2* (A331T) 14258G>A/*MT-ND6* (P139L) | 3505A>G/*MT-ND1* (T67A) 5046G>A/*MT-ND2* (V193I) 15884G>C/*MT-CYB* (A380P) |  | Chronic Periodontitis |

^a^ The mutation 14258G>A/*MT-ND6* is diagnostic of haplogroup H1q3 (<http://www.phylotree.org/tree/R0.htm>). It occurred as a single mutational event in the ancestral H1q3 mitogenome.

^b^ The two HV2a2 mitogenomes most likely harbor the mutation 14258G>A/*MT-ND6* by descent (one mutational event).

^c^ The mutation 14258G>A/*MT-ND6* is diagnostic of haplogroup U3a1a1 (<http://www.phylotree.org/tree/U.htm>). It occurred as a single mutational event in the ancestral U3a1a1 mitogenome.

^d^ The two W3a1b mitogenomes most likely harbor the mutation 14258G>A/*MT-ND6* by descent (one mutational event).

^e^ The two H5b mitogenomes most likely share the mutational motif m.9966G>A/MT-CO3 (V254I), 12033A>G/MT-ND4 (N425S), 14258G>A/MT-ND6 (P139L) by descent. For the mutation 14258G>A/MT-ND6, the single mutational occurrence is further supported by its absence in all other published H5b mitogenomes.
