# Supplementary material for: Peculiar combinations of individually non-pathogenic missense mitochondrial DNA variants cause low penetrance Leber’s hereditary optic neuropathy
Source: PLoS Genet. 2018 Feb 14;14(2):e1007210. doi: 10.1371/journal.pgen.1007210 (PMC5828459; doi:10.1371/journal.pgen.1007210)
Supplement: S6 Table — (DOCX) [file pgen.1007210.s007.docx]

**S6 Table.** Mitogenome sequences carrying the m.10680G>A/*MT-ND4L*, p.A71T, in common databases (a total of 31,787 mitogenomes)

| **Genbank-HGDP-1000 GP ID** | **HmtDB Genome Identifier** | **Haplogroup** | **Private Variants** | | | **Haplogroup Diagnostic Missense Variants** | **Number of Mutational Events**  **m.10680G>A** | **Phenotype** |
| --- | --- | --- | --- | --- | --- | --- | --- | --- |
|  |  |  | **Synonymous** | **Non-Coding** | **Missense** |  |  |  |
| JN866824 | PA_XX_XX_0343 | B4a1e | 3540T>C/*MT-ND1* | 2352T>C/*MT-RNR1* | 10680G>A/*MT-ND4L* (A71T) | 3548T>C/*MT-ND1* (I81T) | 1 | LHON [32] |
| FJ986465 | PA_XX_XX_0331 | B4d1 | 13449C>T/*MT-ND5* 14239C>T/*MT-ND6* | 185G>A/*MT-HV2* 189A>G/*MT-HV2* 489T>C/*MT-HV2* | 10680G>A/*MT-ND4L* (A71T) 14484T>C/*MT-ND6* (M64V) | 13942A>G/*MT-ND5* (T536A) 15038A>G/*MT-CYB* (I98V) | 1 | LHON [26] |
| KF540683 | AS_TW_0178 | D4a8 |  |  | 10680G>A/*MT-ND4L* (A71T) | 5178C>A/*MT-ND2* (L237M) 8414C>T/*MT-ATP8* (L17F) 10400C>T/*MT-ND3* (T114A) 14979T>C/*MT-CYB* (I78T) | 1 | Normal |
| JN866825 | PA_XX_XX_0344 | D6a1 | 8251G>A/*MT-CO2* 15688C>T/*MT-CYB* 11809T>C/*MT-ND4* | 16192C>T/*MT-HV1* | 3745G>A/*MT-ND1* (A147T) 13327A>G/*MT-ND5* (T331A) 10680G>A/*MT-ND4L* (A71T) | 5178C>A/*MT-ND2* (L237M) 10400C>T/*MT-ND3* (T114A) | 1 | LHON [32] |
| JX153117 | EU_GR_0092 | H13a2a | 9824T>C/*MT-CO3* |  | 10680G>A/*MT-ND4L* (A71T) |  | 1 | Normal |
| JQ703337 | EU_NL_0018 | H1b | 13389C>T/*MT-ND5* |  | 10680G>A/*MT-ND4L* (A71T) |  | 1 | Normal |
| JQ702415 | XX_XX_1893 | H1 | 4742T>C/*MT-ND2* 8865G>R/*MT-ATP6* | 16075T>C/*MT-HV1* 16189T>C/*MT-HV1* 16224T>C/*MT-HV1* 16319G>A/*MT-HV1* 16324T>C/*MT-HV1* | 10680G>A/*MT-ND4L* (A71T) |  | 1 | Normal |
| JQ704194 | XX_XX_2693 | H1t | 3447A>G/*MT-ND1* 6167T>C/*MT-CO1* 6293T>C/*MT-CO1* | 2141T>C/*MT-RNR2* | 9911C>A/*MT-CO3* (F235L) 10680G>A/*MT-ND4L* (A71T) |  | 1 | Normal |
| KF450878/ HGDP00119 | XX_XX_6433 | HV | 3351C>T/*MT-ND1* 3744A>G/*MT-ND1* 4829A>G/*MT-ND2* 11566A>G/*MT-ND4* 12630G>A/*MT-ND5* 13437T>C/*MT-ND5* | 16158A>T/*MT-HV1* | 10680G>A/*MT-ND4L* (A71T) |  | 1 | Normal |
| KJ445748/ HGDP00621 | XX_XX_4630 | L0a1b1a |  | 593T>C/*MT-TF* | 3311C>T/*MT-ND1* (P2L) 10680G>A/*MT-ND4L* (A71T) | 5442T>C/*MT-ND2* (F325L) 5460G>A/*MT-ND2* (A331T) 5911C>T/*MT-CO1* (A3V) 8566A>G/*MT-ATP6* (I14V) 15431G>A/*MT-CYB* (A229T) | 1 | Normal |
| KJ669103 | AF_NA_0197 | L0k1a2a | 3876A>G/*MT-ND1* |  | 10680G>A/*MT-ND4L* (A71T) | 5442T>C/*MT-ND2* (F325L) 7257A>G/*MT-CO1* (I452V) 9136A>G/*MT-ATP6* (I204V) 10920C>T/*MT-ND4* (P54L) 13819T>C/*MT-ND5* (F495L) 13928G>C/*MT-ND5* (S531T) | 1 | Normal |
| GU377087 | PA_XX_XX_0306 | M13a1b | 9053G>A/*MT-ATP6* 10646G>A/*MT-ND4L* | 980T>C/*MT-RNR1* 16239C>T/*MT-HV1* 16391G>A/*MT-HV1* | 10680G>A/*MT-ND4L* (A71T) | 3644T>C/*MT-ND1* (A113A) 6253T>C/*MT-CO1* (M117T) 9053G>A/*MT-ATP6* (S176N) 10400C>T/*MT-ND3* (T114A) 13135G>A/*MT-ND5* (A267T) | 1 | LHON [31] |
| JF742198 | AS_NP_0020 | M33b2 | 8802T>C/*MT-ATP6* 15326G>A/*MT-CYB* 15514T>C/*MT-CYB*  15868C>T/*MT-CYB* | 1438G>A/*MT-RNR1* 3202T>C/*MT-RNR2* | 3469C>T/*MT-ND1* (L55F) 6261G>A/*MT-CO1* (A120T) 10680G>A/*MT-ND4L* (A71T) | 10400C>T/*MT-ND3* (T114A) | 1 | Normal |
| KF162184 | EU_DK_1441 | R9b1a3 |  |  | 10680G>A/*MT-ND4L* (A71T) | 3316G>A/*MT-ND1* (A4T) 13928G>C/*MT-ND5* (S531T) | 1 | Normal |
