# Supplementary figures and images for: Peculiar combinations of individually non-pathogenic missense mitochondrial DNA variants cause low penetrance Leber’s hereditary optic neuropathy
Source: PLoS Genet. 2018 Feb 14;14(2):e1007210. doi: 10.1371/journal.pgen.1007210 (PMC5828459; doi:10.1371/journal.pgen.1007210)

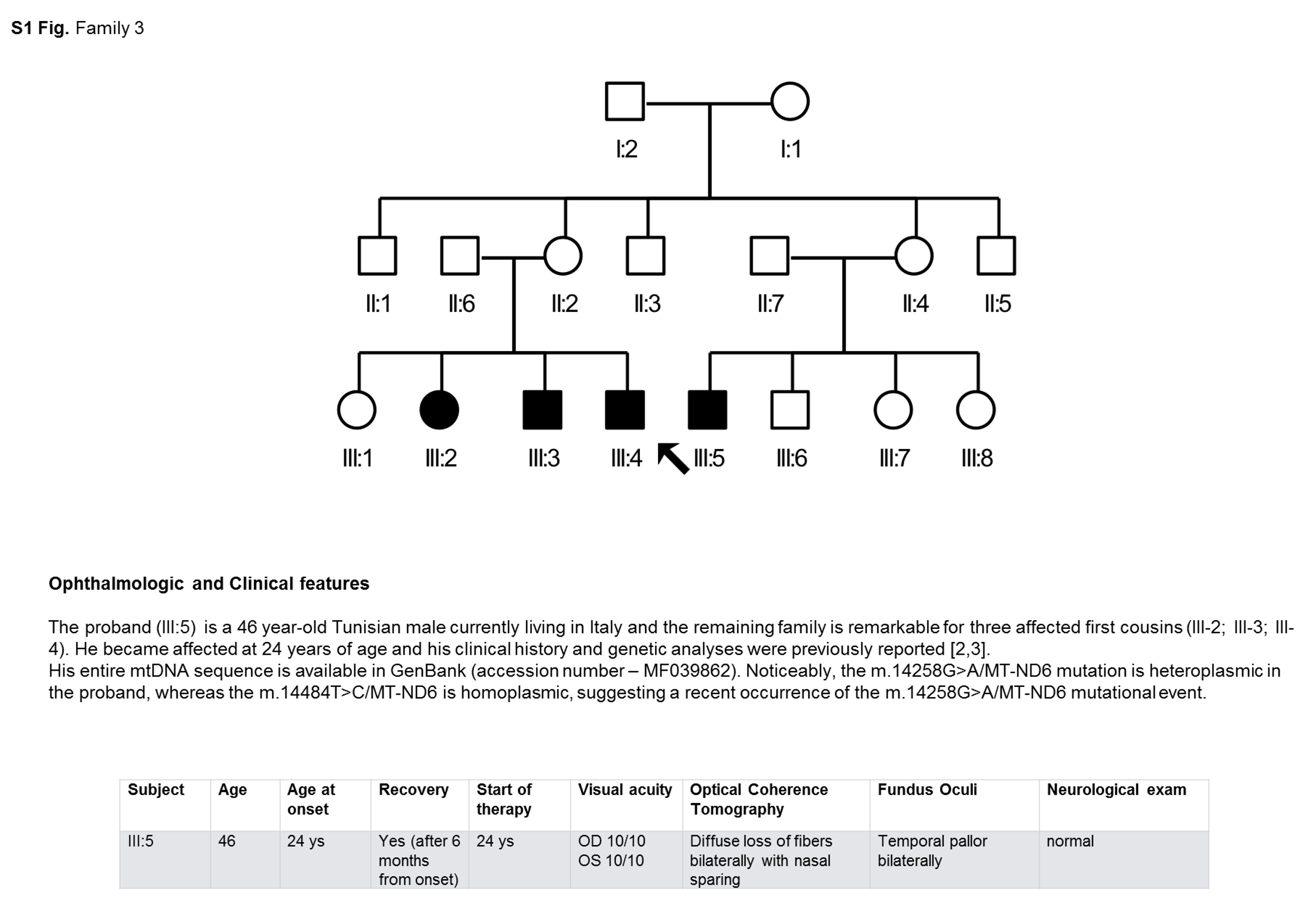

Supplement: S1 Fig — (TIF) [file pgen.1007210.s008.tif]

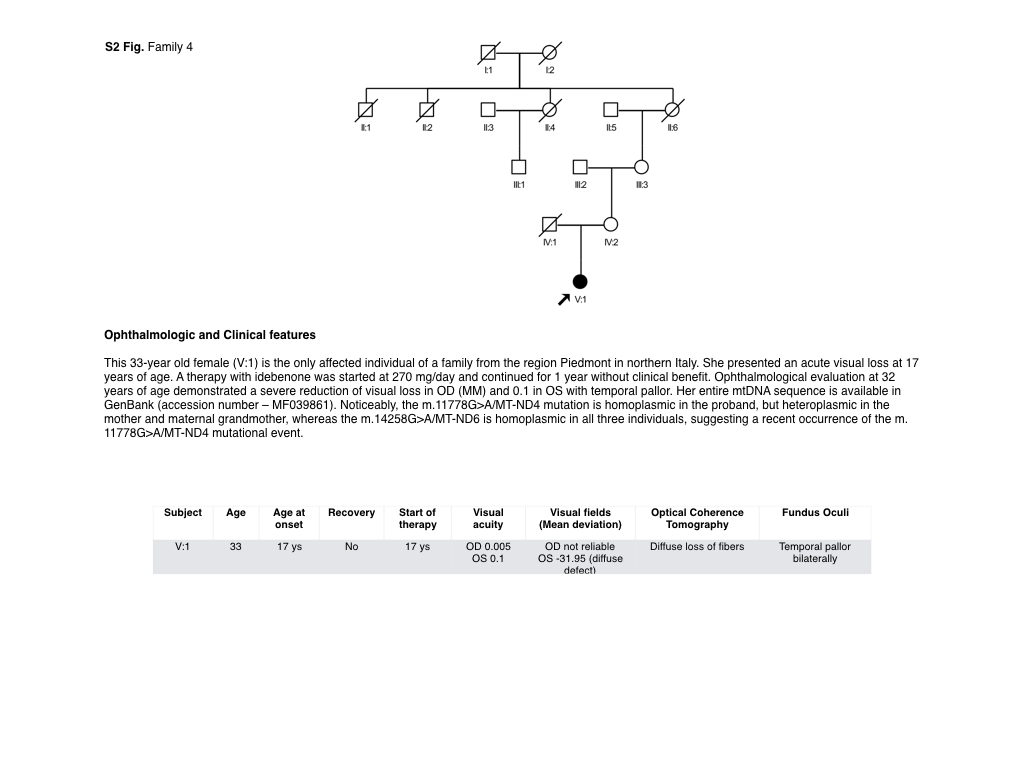

Supplement: S2 Fig — (TIFF) [file pgen.1007210.s009.tiff]

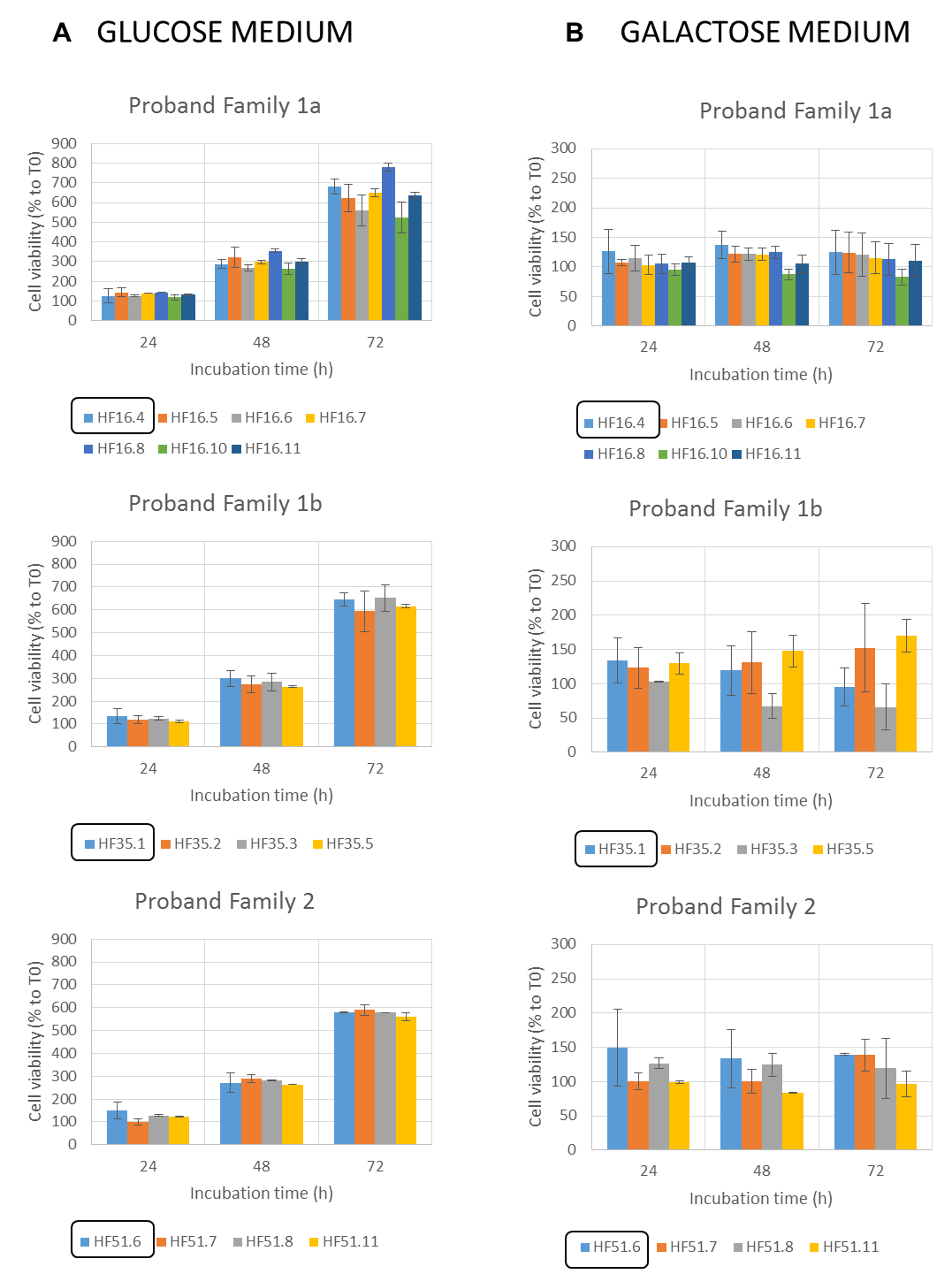

Supplement: S3 Fig — A. Cell viability in complete medium (25 mM glucose) for different times (0, 24, 48, 72h). Data are expressed as percentage of T0 (n = 3; mean ± SD). B. Cell viability in galactose (5 mM) medium for different times (0, 24, 48, 72h). Data are expressed as percentage of T0 (n = 3; mean ± SD). (TIF) [file pgen.1007210.s010.tif]

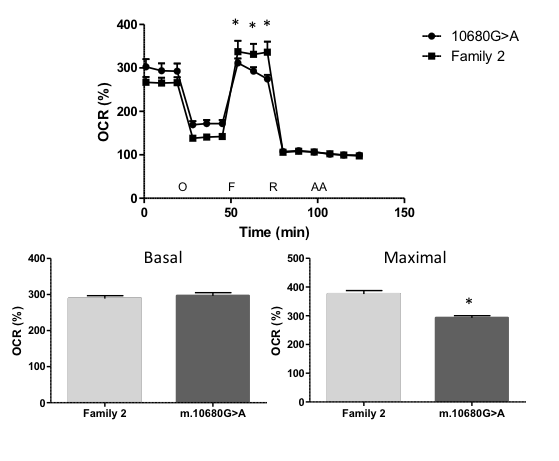

Supplement: S4 Fig — OCR traces as pmol O2/min, after the injection of 1μM oligomycin (O), 0.2μM FCCP (F), 1μM rotenone (R) and 1μM antimycin A (AA) (mean ± SD). Asterisks indicate statistical significance (n = 3; * p<0.05). (TIF) [file pgen.1007210.s011.tif]

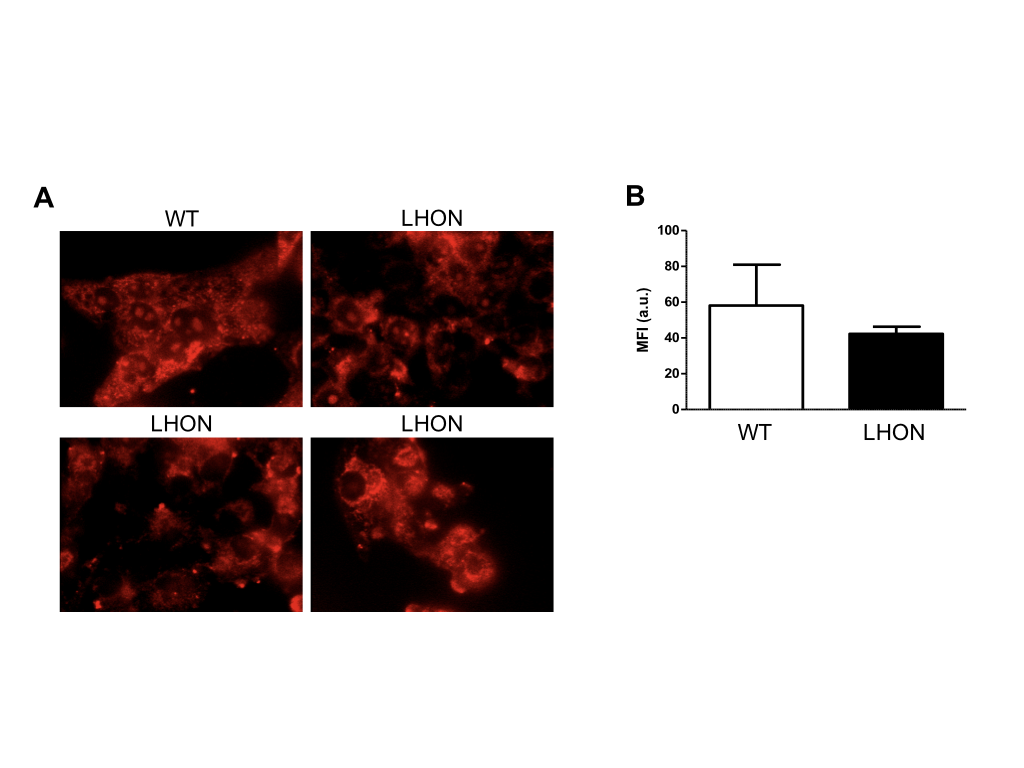

Supplement: S5 Fig — A. Mitochondrial superoxide anion production determined by epifluorescence microscopy using MitoSOX fluorescent dye. Cells were visualized with a digital imaging system, using an inverted epifluorescence microscope (magnification x63/1.4 oil objective) at 580nm. Images are representative of 3 different experiments. B. Hydrogen peroxide levels were measured using H2DCFDA by flow cytometry, as described in materials and methods. Data are mean ± SD (n = 3). (TIFF) [file pgen.1007210.s012.tiff]

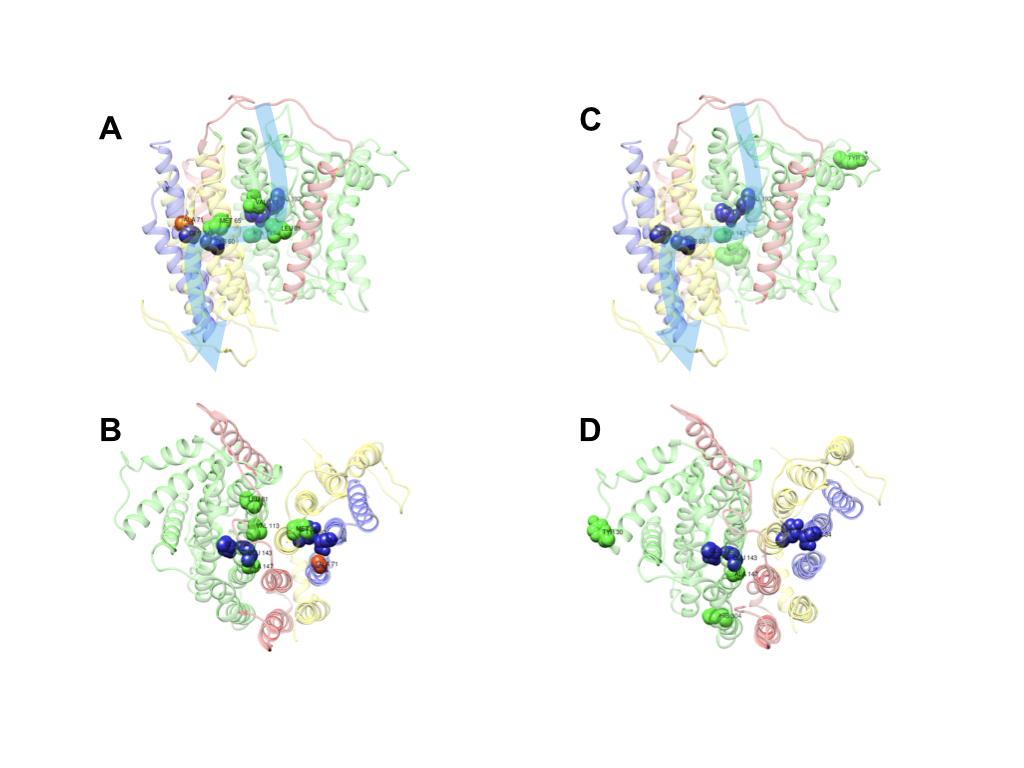

Supplement: S6 Fig — Positioning of the combinations of variants in three LHON Chinese families (A-B) [26,31,32] and the adaptive variants for high altitude in Tibet (C-D) [27,42] on structure of ovine CI obtained by cryo-EM [34], using the UCSF Chimera software. In panels A and B, the ovine amino acid Ala71 (corresponding to human p.A71T, m.10680G>A/MT-ND4L) is shown as red labelled sphere, and this variant is associated in each family with Met65 (corresponding to human p.M64V, m.14484T>C /MT-ND6), Ala147 (corresponding to human p.A147T, m.3745G>A/MT-ND1), Leu81 (corresponding to human p.I81T, m.3548T>C/MT-ND1) or Val113 (corresponding to human p.V113A, m.3644T>C/MT-ND1), shown as green labelled spheres. In panels C and D, the ovine amino acids Ala147 (corresponding to p.A147T, m.3745G>A/MT-ND1), His304 (corresponding to human p.Y139H, m.4216T>C/MT-ND1) and Tyr30 (corresponding to human p.Y30H, m.3394T>C/MT-ND1) are shown as green labelled spheres. In all panels, residues Glu143/ND1, Glu192/ND1, Glu34/ND4L, Tyr60/ND6, the key residues for the E-channel (near Q-site), are shown as blue labelled spheres [27]. The backbones of ND1, ND4L, ND6 and ND3 are shown as ribbons, in green, blue, yellow and red, respectively. The variants combination in LHON Chinese families (A-B) and adaptive variants for high altitude in Tibet (C-D) are displayed as front (A-C) and upper (B-D) views. Light blue arrows indicate the proposed proton translocation pathway. (TIFF) [file pgen.1007210.s013.tiff]
